# *In silico* guided reconstruction and analysis of ICAM-1-binding *var* genes from *Plasmodium falciparum*

**DOI:** 10.1038/s41598-018-21591-8

**Published:** 2018-02-19

**Authors:** Eilidh Carrington, Thomas D. Otto, Tadge Szestak, Frank Lennartz, Matt K. Higgins, Chris I. Newbold, Alister G. Craig

**Affiliations:** 1 Liverpool School of Tropical Medicine, Pembroke Place, Liverpool, L3 5QA UK; 2 Wellcome Trust Sanger Institute, Wellcome Genome Campus, Hinxton, Cambridge CB10 1SA UK; 3 Department of Biochemistry, University of Oxford, South Parks Road, Oxford, OX1 3QU UK; 4 Weatherall Institute of Molecular Medicine, University of Oxford, John Radcliffe Hospital, Headington, Oxford OX3 9DS UK; 5 Present Address: Malaria Gene Regulation Lab, Swiss Tropical and Public Health Institute, Socinstrasse 57, 4051 Basel, Switzerland; 6 Present Address: Institute of Infection, Immunity and Inflammation, University of Glasgow, College of Medical, Veterinary and Life Sciences, Sir Graeme Davies Building, 120 University Place, Glasgow, G12 8TA UK

## Abstract

The *Plasmodium falciparum* variant surface antigen PfEMP1 expressed on the surface of infected erythrocytes is thought to play a major role in the pathology of severe malaria. As the sequence pool of the *var* genes encoding PfEMP1 expands there are opportunities, despite the high degree of sequence diversity demonstrated by this gene family, to reconstruct full-length *var* genes from small sequence tags generated from patient isolates. To test whether this is possible we have used a set of recently laboratory adapted ICAM-1-binding parasite isolates to generate sequence tags and, from these, to identify the full-length PfEMP1 being expressed by them. In a subset of the strains available we were able to produce validated, full-length *var* gene sequences and use these to conduct biophysical analyses of the ICAM-1 binding regions.

## Introduction

The pathology of severe *Plasmodium falciparum* malaria is not simple and is thought to involve the ability of erythrocytes infected with this parasite to bind to host endothelial cells lining the microvasculature^1^. The protein responsible for most of this adhesion is the variant surface antigen *Plasmodium falciparum* erythrocyte membrane protein 1 (PfEMP1). This is encoded by a family of approximately 60 *var* genes per parasite genome with limited overlap of the *var* gene repertoires between isolates^2–4^. As PfEMP1 can be targeted by the host immune system, it undergoes a complex process of switching transcription between *var* gene family members^5^ resulting in (mostly) mutually exclusive expression of a single member of the family on the surface of the infected erythrocyte. Consistent with their role in avoiding immune clearance, the extracellular PfEMP1 sequences are highly variable consisting of DBL and CIDR regions that vary in their number, position and composition. Several studies have sought to relate motifs from PfEMP1s to aspects of malaria disease using *var* gene upstream sequences^6^, the relatively conserved N-terminal DBL1 domain^7^ and a more complex categorization of the combination of specific DBL and CIDR domain types in ‘domain cassettes’^4^. All of these have shown associations between classes of PfEMP1s and disease, most notably the upsA types and CIDR1α^8,9^.

Linked to the variable nature of PfEMP1 has been the expectation that the diversity in sequence and DBL/ CIDR composition would translate into variable adhesion characteristics of erythrocytes infected with parasites expressing different *var* genes. This is seen in several laboratory-adapted isolates for which the full-length sequences of the expressed PfEMP1s are known and regions of PfEMP1s have been mapped to specific binding behaviours^1,10^, revealing a wide range of potential receptors on host cells. However, it has been more challenging to link binding to a specific receptor with disease, with quite diverse results being reported, except for placental malaria and CSA^11^, and in the more recent case of binding to Endothelial Protein C Receptor (EPCR)^12^. Despite these difficulties, we are beginning to understand more about the functions of specific regions of PfEMP1, in particular the role of defined domains in binding to specific host receptors (e.g. CIDRα1 to EPCR; some DBLβ to ICAM-1) and thereby developing algorithms to understand the potential binding characteristics of a PfEMP1 variant from its sequence alone^13^. The challenge running in parallel with this is how to obtain the full-length PfEMP1 sequences from patient samples that may be limited in quantity.

There are molecular techniques to identify and sequence *var* genes but these are labour intensive and not suited to anything beyond limited throughput^14^. However, as more *var* gene sequences have become available in genome databases, while the diversity of the gene family is, as expected, very high, there are significant overlaps in *var* gene sequences coming from divergent parasite isolates. This latter finding suggests that it may be possible to look for existing *var* gene sequences for almost any PfEMP1 in the sequence databases and to use this information to provide a framework to generate *de novo* PfEMP1 full-length data from small sequence tags produced by RT-PCR from patient samples. To test whether this approach is feasible, we used a set of recently laboratory-adapted ICAM-1-binding isolates to see whether we could identify the full-length *var* gene sequences being expressed and then to use these to characterise their respective PfEMP1 binding characteristics.

## Results

### Identification of dominantly expressed var genes of culture-adapted patient isolates

**Figure 1:**
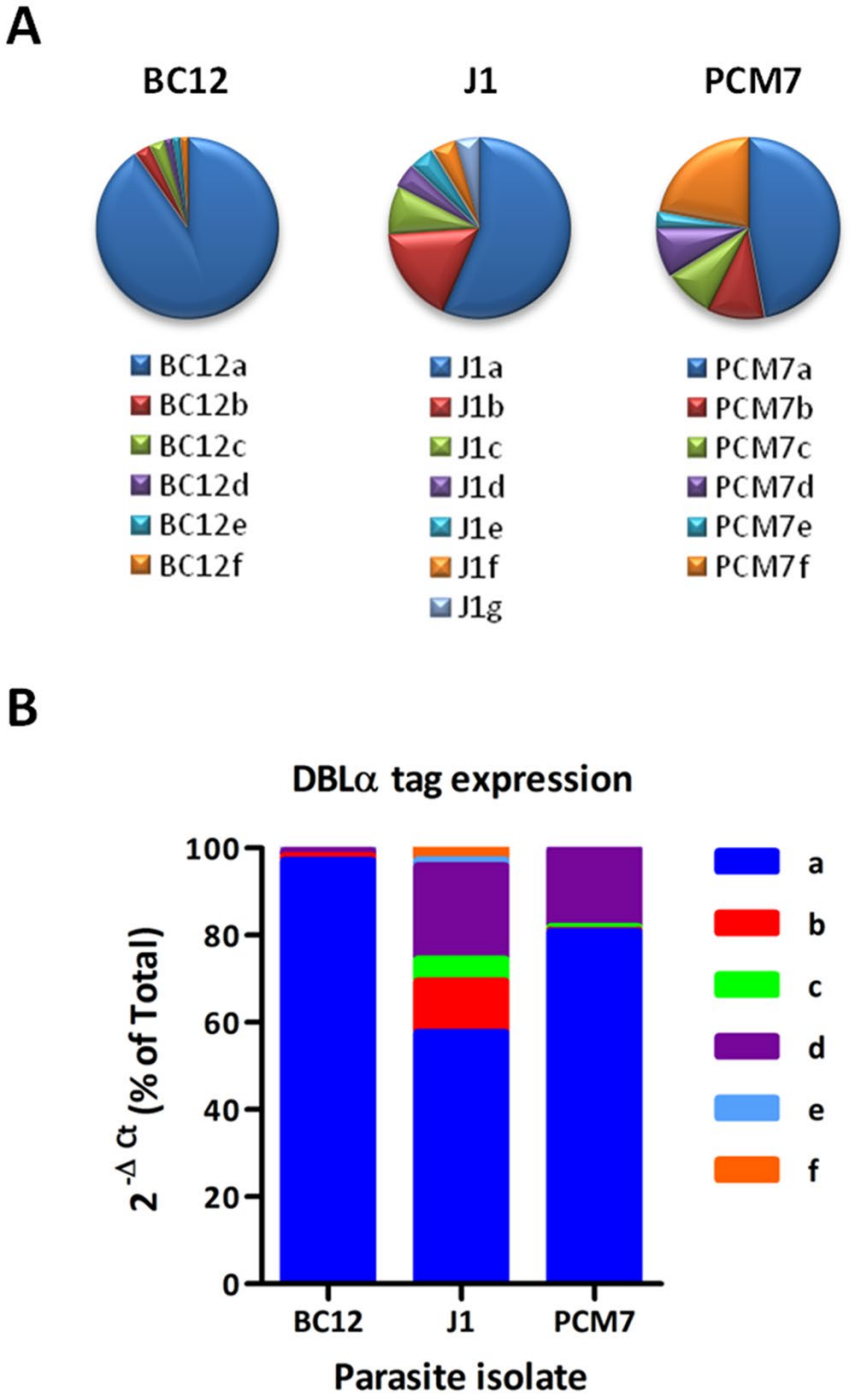
Identification of expressed DBLα tags of three ICAM-1 selected culture-adapted patient isolates. (**A**) Expressed DBLα tags were identified by RT-PCR of ICAM-1 selected parasite isolates BC12, J1 and PCM7, cloned and 24 tags from each sequenced by Sanger sequencing. Unique tags were named a, b, c, etc for each strain. PCM7f refers to a set of unique DBLα tags found only once in the cloning experiments, for which qPCR was not performed. (**B**) RT-qPCR was carried out with primers specific to each DBLα tag identified in A. Ct values were normalised against the ASL internal control gene and are shown as percentage of total for each cDNA. Results from three different RNA extractions for BC12 and PCM7 and a single RNA extraction for J1 are shown.

In order to identify novel *var* genes that mediate binding to ICAM-1, three ICAM-1-selected, culture-adapted patient isolates – BC12, J1 and PCM7 – were re-selected on ICAM-1 and returned to culture for expansion before RNA extraction and cDNA synthesis. Expressed *var* genes were identified by PCR amplification of homology regions in the DBLα domain using universal primers^15^ to produce short sequence tags known as DBLα tags. Twenty-four DBLα tags from each culture-adapted patient isolate were cloned, sequenced and named alphabetically in order of cloning frequency. The number of unique sequences varied between isolates but all contained a prominent DBLα tag that was cloned at a high frequency, namely BC12a, J1a and PCM7a in isolates BC12, J1 and PCM7, respectively (Fig. 1A). Reverse transcriptase quantitative PCR (RT-qPCR), carried out with tag-specific primers designed against regions of variable sequence within the DBLα tag, confirmed that the most frequently cloned DBLα tag was in fact the most highly expressed tag for each isolate (Fig. 1B). DBLα tags J1b and J1d, from isolate J1, and PCM7d, from isolate PCM7, were also expressed at a relatively high level (Fig. 1B), suggesting they may also have a role in mediating ICAM-1-binding.

### Reconstruction of full length var genes using the Pf3k var gene database

Sequencing *var* genes *de novo* is challenging due to their size (4.5–10 kb) and sequence composition which is highly variable with short regions of homology^3,4^. Here, we tested whether searching the only comprehensive *var* gene database for a match to a short DBLα tag can predict the full length *var* gene sequence. The highly expressed DBLα tags of BC12, J1 and PCM7 coming from the qRT-PCR analysis were BLAST searched against the Pf3k *var* gene database with parameters of at least 99% identity and 95% overlap. Five of the six DBLα tags resulted in ≤10 hit sequences each, whereas J1a resulted in 153 hits (Table 1). The sequences obtained from the tags have been submitted to Genbank (# MG220391 – MG220396 – Table 1).

**Table 1:** Number of hits to each DBLα tag in the Pf3k *var* gene database detected by BLAST search.

DBLα tag	Accession number	# database hits
BC12a	MG220391	2
J1a	MG220392	153
J1b	MG220393	1
J1d	MG220394	3
PCM7a	MG220395	4
PCM7d	MG220396	10

The accession numbers refer to the extended *var* gene sequence for each tag.

The two returned sequences to BC12a share 99% sequence identity (based on measuring similarity across the whole length of the returned sequences and not restricted to the DBLα tag) and are therefore essentially the same gene. Similarly, the respective returned sequences to J1d and PCM7a, share 99% sequence identity within each group. Sequence hits to PCM7d (N = 10) shared 97–99% identity, with one hit containing an additional ~200 bp insertion. The 153 hits to J1a share sequence similarity over ~3 kb at the 5′ end but are variable downstream. Therefore, it appears that the more sequence hits that are returned from the DBLα tag search, the more likely it is that they will be of varying full-length sequence, at least in our small sample set.

**Figure 2:**
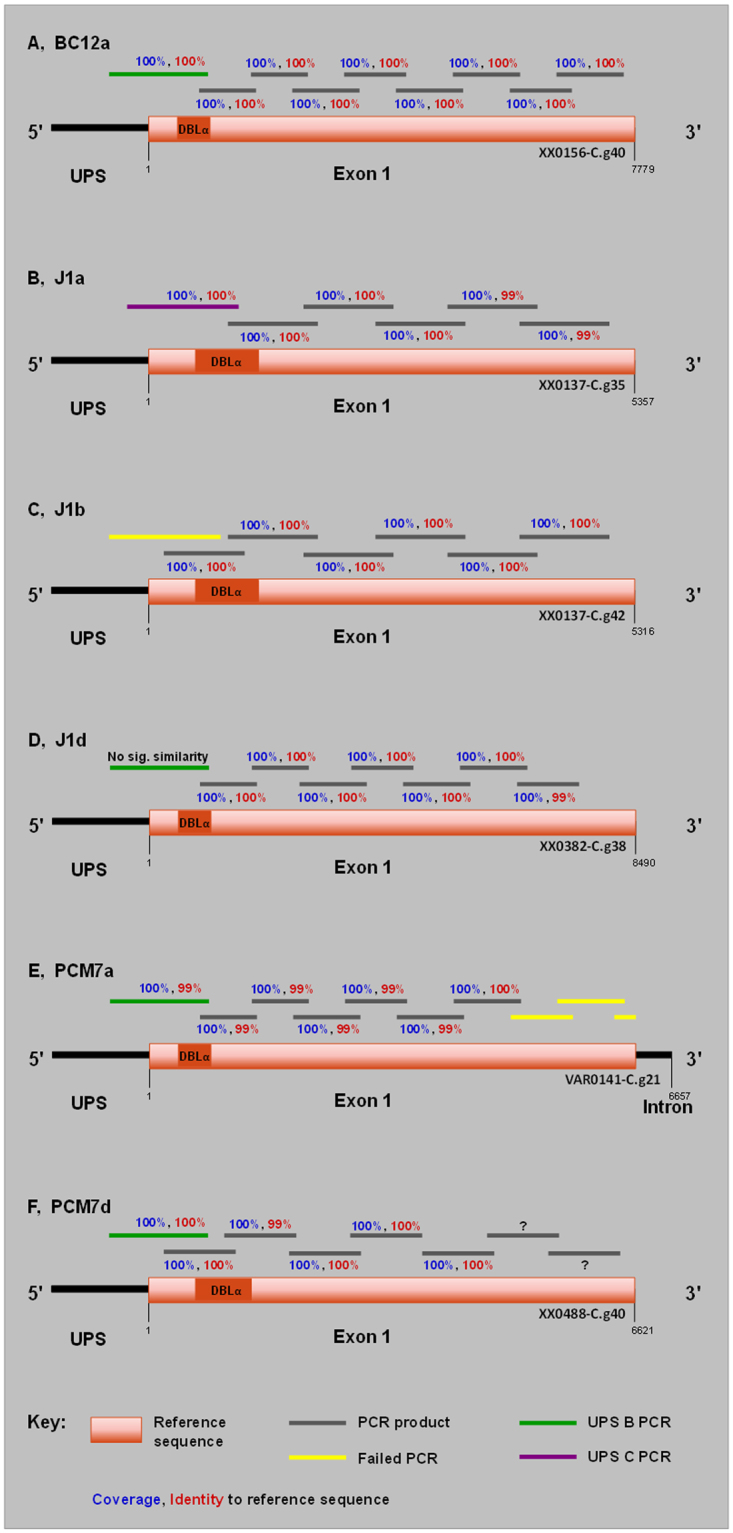
Schematic of *var* gene reconstruction using Pf3k *var* gene database matches to newly identified DBLα tags. The highly expressed DBLα tags of BC12 (**A**), J1 (**B**–**D**) and PCM7 (**E**,**F**) were BLAST searched against the Pf3k *var* gene database. Resulting matches were treated as reference sequences (orange) for PCR primer design resulting in ~1 kb overlapping products amplified from genomic DNA, represented by grey bars. PCR products were cloned, sequenced and compared to their respective reference sequences, with percentage coverage (blue) and identity (red) shown. Upstream typing was also performed using UPS A, B and C specific primers^6^ in combination with a tag-specific primer. Sequence similarity of UPS fragments to that of the coding reference sequence is shown. Failed PCRs are shown in yellow. Question marks in F denote amplified products of highly AT-rich sequence, suggesting priming occurred within an intron. All primer sequences are listed in Table S1.

Returned sequence hits to each DBLα tag were chosen as reference sequences for primer design based on length and consensus sequence. Primers were designed every ~1 kb to produce overlapping products which were cloned and sequenced. In addition, we performed 5′ upstream (UPS) typing using primers specific for UPS types A, B and C^6^ which revealed BC12a, J1d, PCM7a and PCM7d to be UPS B type and J1a UPS C type. We were unable to UPS type J1b. BC12a, J1a and J1b fragments shared ≥99% identity with their respective reference sequences (Fig. 2A,B and C). J1d fragments downstream of the DBLα tag shared ≥99% identity with its reference sequence but the UPS fragment (J1d_1-567) had no significant similarity in the coding region (Fig. 2D). PCM7a fragments amplified across three quarters of the length of the reference gene with ≥99% identity but the remaining fragments failed to amplify (Fig. 2E). PCM7d fragments also shared ≥99% identity with their reference sequence with the exception of two 3′ fragments whose sequences were highly AT rich, suggesting that priming occurred within the intron (Fig. 2F, denoted by question marks). A conserved primer to exon 2^16^ was used to fully sequence PCM7a and J1b. However, we were unable to gain the complete exon 1 sequence of genes BC12a, J1a, J1d and PCM7d due to either multiple products of the exon 2 PCR or cloning difficulty (data not shown).

### Definition of PfEMP1 domain structure by sequence analysis

**Figure 3:**
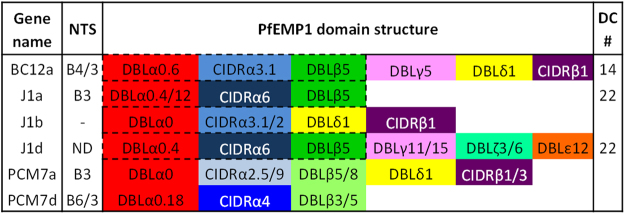
PfEMP1 domain and DC annotation of *var* genes reconstructed using the Pf3k database. Domain boundaries were defined by the VarDom 1.0 Server and individual domains BLAST searched against the 7 genomes dataset in Rask *et al*.^4^. The top hits were analysed and domain subtypes identified based on consensus sequence and coverage. Where multiple subtypes were identified, both are shown if they are of equal standing (e.g. 0.4/12) or excluded if more than two subtypes displayed similar coverage. Dashed lines outline domain cassettes (DC) with the DC number shown in the far-right column. ND: not defined, no matches by BLAST search.

The domain composition of PfEMP1 proteins determines host receptor binding, with different domain types and subtypes known to mediate binding specificity. We analysed the reconstructed *var* gene sequences to obtain their PfEMP1 domain structure. Domain boundaries and types were defined by the VarDom 1.0 Server and each domain was BLAST searched against the 7 genomes data set^4^ to identify subtypes. Domain structures are represented schematically in Fig. 3. All are of DBLα type 0 with varying sub-classifications, a type almost exclusively associated with UPS B and C *var* genes^4^, concordant with our UPS typing. Similarly, the six genes analysed all contain CIDRα subtypes predicted to bind CD36, a phenotype of UPS B and C type *var* genes^17^. BC12a, J1a and J1d contain DBLβ5 which is predicted to mediate binding to ICAM-1^18,19^. The DBLβ domains of PCM7a and PCM7d had BLAST hits to two different domain subtypes – PCM7a: 5/8 and PCM7d: 3/5. Three DBLβ3 domains tested have been shown to bind ICAM-1 but several others do not^18–20^. Interestingly, J1b does not contain a DBLβ domain, suggesting that it is not involved in ICAM-1 binding. The J1b DBLα tag represented 12% of *var* gene expression in the J1 isolate (Fig. 1B) and may be the result of a switching event after ICAM-1 selection.

### Sequence to phenotype analysis of DBLβ domains predicted to bind ICAM-1

**Figure 4:**
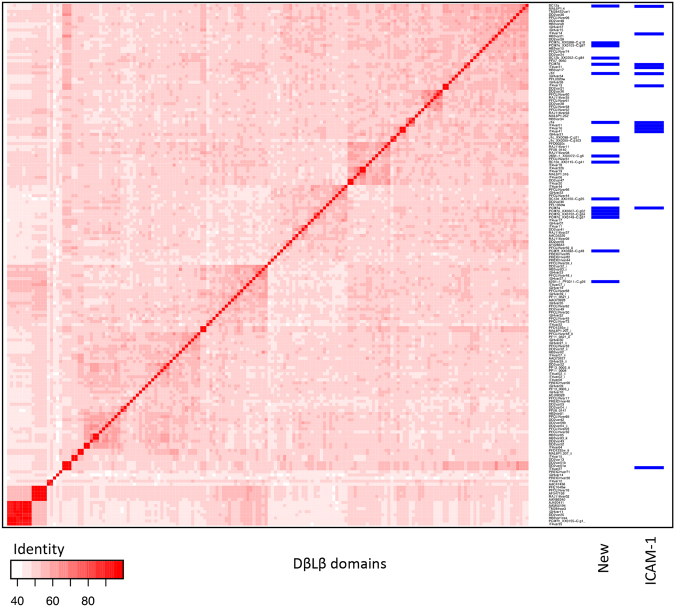
Blast distance matrix of DBLβ domains. Visualization of all-against-all blast of all DBLβ domains described in the 7 genomes dataset^4^ and the DBLβ domains identified in this study from ICAM-1 binding patient isolates BC12, J1 and PCM7. The two columns of blue barcodes show the clustering of the newly obtained DBLβ domains and the known ICAM-1 binding domains including the DBLβ domains of the dominantly expressed *var* genes of BC12, J1 and PCM7.

We analysed the 5 newly identified DBLβ domains of the ICAM-1 binding patient isolates in more detail by comparing them to known DBLβ sequences. Due to the highly recombinogenic nature of *var* genes, phylogenetic trees do not adequately capture the evolutionary history of DBL domains. We therefore chose to carry out a global sequence similarity approach by constructing a blast distance matrix of all DBLβ domains identified in the Rask *et al*. dataset^4^ together with the newly identified sequences obtained here from BC12a, J1a, J1d, PCM7a and PCM7d. The results demonstrate the diverse nature of the DBLβ domains (Fig. 4) with no specific clustering of the ICAM-1 binding domains.

**Figure 5:**
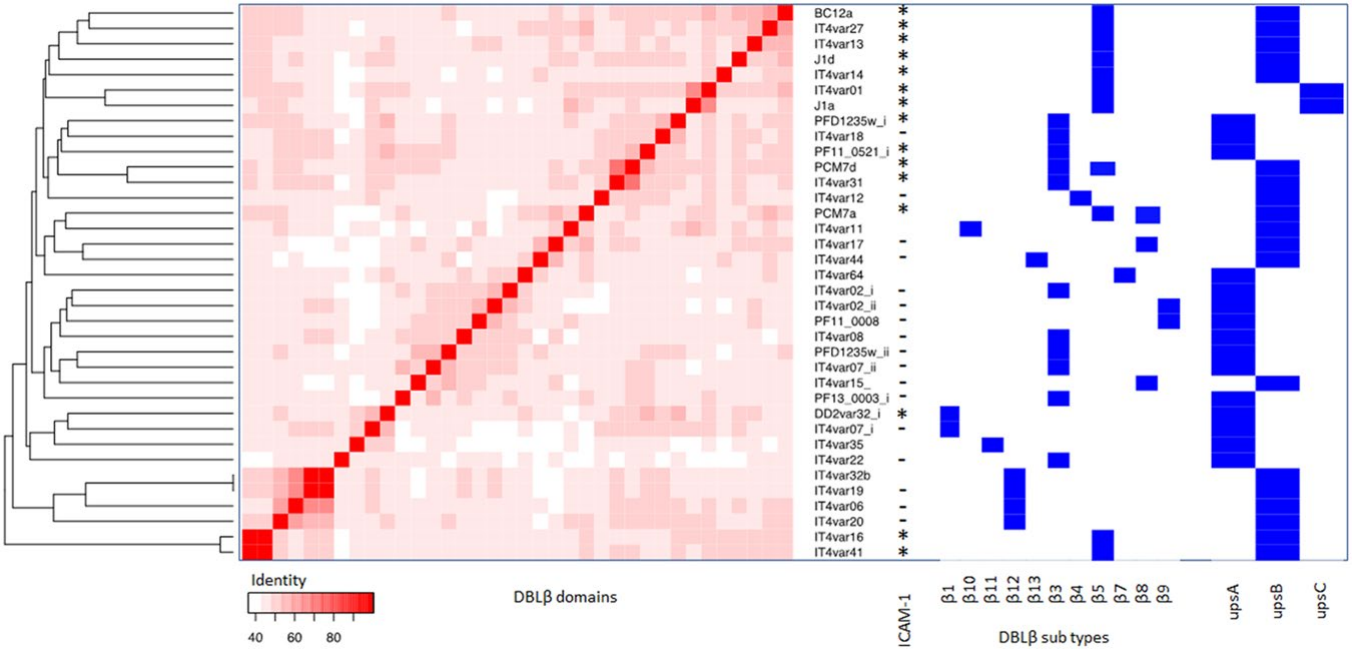
Blast distance matrix of newly identified DBLβ domains and those previously tested for ICAM-1 binding. Visualization of an all-against-all blast of all IT4 DBLβ domains, five DBLβ domains from 3D7 and one from Dd2 tested for ICAM-1 binding and the dominantly expressed DBLβ domains identified in this study. To each domain we associated annotation on the right, ICAM-1 binding, DBLβ types and the UPS types (as from the VarDom 1.0 Server). ICAM-1 binding domains are highlighted within the columns ICAM-1 binder with a *, non-binders with a - and those not tested for ICAM-1 binding is empty^18–20,39^. UPS type is indicated in boxes at branch ends, along with the DBLβ type.

The majority of DBLβ binding studies have been carried out on the IT4 parasite isolate. Therefore, a second comparison was performed that was restricted to our newly identified DBLβ domains together with those from IT4^18^, five from the 3D7 clone and one from the Dd2 clone that have been tested for ICAM-1 binding (Fig. 5). In this more limited comparison, 12/15 of the ICAM-1 binders cluster very well together at the top of the figure. Of the three that do not, ITvars16 and 41 are next to each other but as far as possible from the main cluster and Dd2var32 appears alone in the middle. 10/15 of the ICAM-1 binding domains are of the UPS B type, three are UPS A and two are UPS C. Overall, it appears that within this more limited group, sequence similarity is an important contributor to ICAM-1 specificity.

### Confirmation of ICAM-1 binding of BC12a and J1a recombinant DBLβ domains

**Figure 6:**
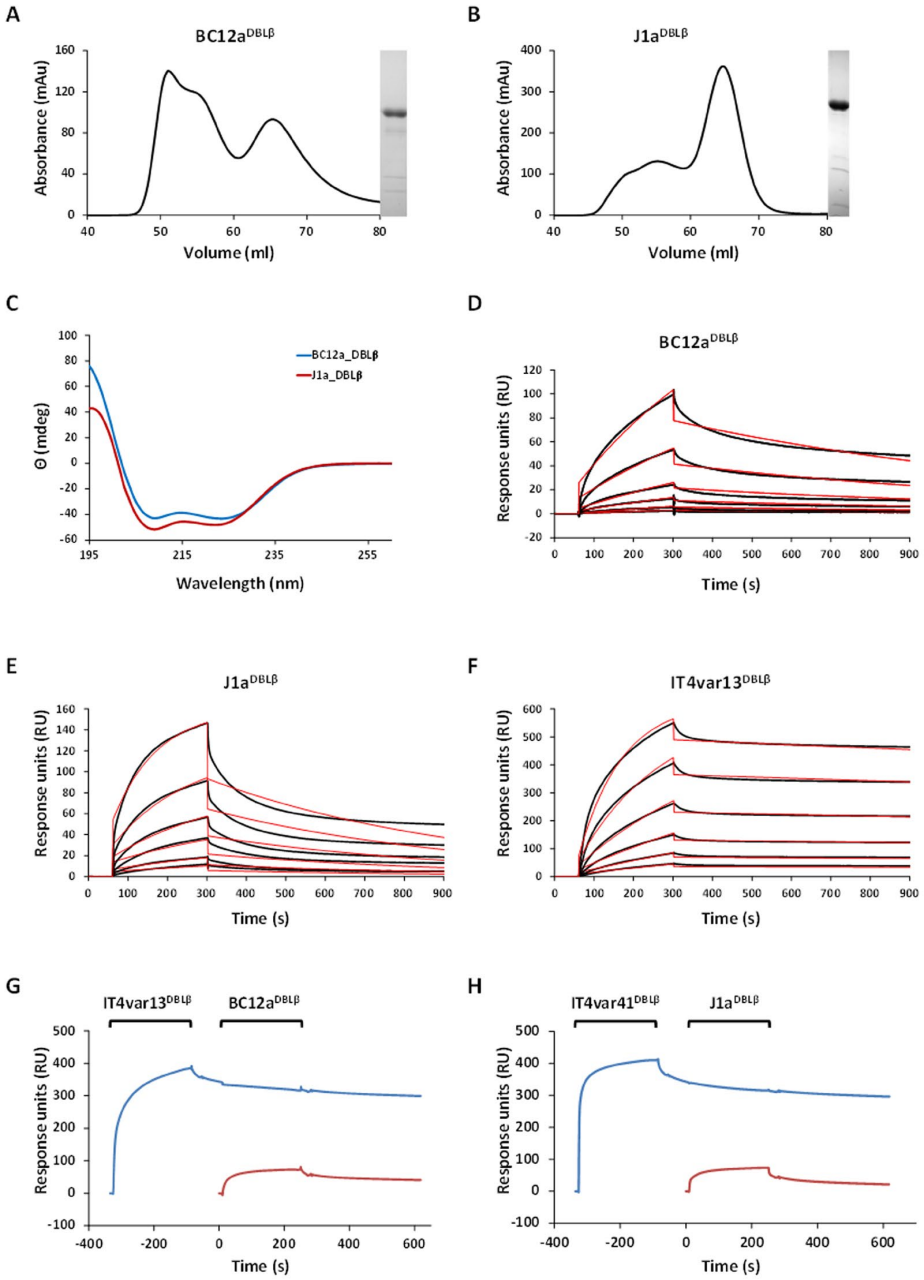
DBLβ domains of BC12a and J1a bind ICAM-1^D1D5^ with nanomolar affinity. Size exclusion chromatographs and purity by SDS-PAGE are shown for BC12^DBLβ^ (**A**) and J1a^DBLβ^ (**B**). The majority of BC12^DBLβ^ is present in aggregated form with a small proportion of monomeric protein (**A**). J1a^DBLβ^ is predominantly monomeric with some aggregated protein evidenced by presence of a large shoulder (**B**). mAu, miliabsorbance units. The purified monomeric samples used in the SPR studies are shown in the gels to the right of the chromatographs in (**A**) and (**B**), with full gels shown in Fig. S1 (BC12) and Fig. S2 (J1a). (**C**) CD analysis of secondary structure. CD spectra were recorded between 195 and 260 nm at 20 °C. For each protein, four measurements were averaged and corrected for buffer absorption. D-F, ICAM-1^D1D5^ was coupled to a sensor chip surface (1100 RU) and DBLβ domains were injected at 30 µl/min with an association time of 240 seconds and a dissociation time of 600 seconds. Shown are sensorgrams for the binding to ICAM-1^D1D5^ of BC12a^DBLβ^ (**D**), J1a^DBLβ^ (**E**) and IT4var13^DBLβ^ (**F**). Data (black lines) are modelled to a 1:1 global interaction model (red lines). Shown are sensorgrams for the sequential binding of IT4var13^DBLβ^ and BC12a^DBLβ^ to ICAM-1^D1D5^ (**G**) and IT4var41^DBLβ^ and J1a^DBLβ^ to ICAM-1^D1D5^ (**H**), blue lines. The response to the second domain alone is shown in red. Data for Fig. 6 is shown in S2–S4 Data.

BC12a and J1a DBLβ domains were expressed in an *E. coli* expression system and purified to homogeneity (Fig. 6A and B). Correct protein folding was confirmed by circular dichroism spectroscopy showing the presence of a predominantly α-helical protein, as expected for a DBL domain^21^ (Fig. 6C). Surface plasmon resonance (SPR) was used to test binding of BC12a^DBLβ^ and J1a^DBLβ^ to ICAM-1^D1D5^ and obtain kinetic parameters of the interaction. We included the previously characterised IT4var13^DBLβ^ ^22^ for comparison. All SPR experiments were carried out using purified monomeric fractions shown in Fig. 6A and B. Binding kinetics of IT4var13^DBLβ^ to ICAM-1^D1D5^ display a fast association rate and a slow dissociation rate, with a K_D_ of 3.45 nM (Fig. 6F and Table 2), comparable to published values for this domain^22^. BC12a^DBLβ^ and J1a^DBLβ^ both bind to ICAM-1^D1D5^ with mid-nanomolar affinities of 197 nM and 51.7 nM respectively (Table 2). While these affinities are lower than those of IT4var13^DBLβ^, due to slower on-rates and faster off-rates, this variability in affinity of PfEMP1 domains for a single ligand is indeed often seen in the case of other domains that bind to ICAM-1, EPCR or CD36^5,22–24^.

**Table 2:** Kinetic parameters derived from SPR experiments.

Interaction	k_a_ (X 10^4^ M^−1^ s^−1^)	k_d_ (X 10^−4^ s^−1^)	K_D_ (nM)
ICAM-1 ^D1D5^:: BC12a^DBLβ^	0.48	9.44	197
ICAM-1 ^D1D5^:: J1a^DBLβ^	2.96	15.3	51.7
ICAM-1 ^D1D5^:: IT4var13^DBLβ^	3.61	1.25	3.45

We also used an SPR competition assay to test whether the DBLβ domains have an overlapping binding surface. This involves sequential injection of DBLβ domains over the ICAM-1^D1D5^ surface with the expectation that if the domains have separate binding surfaces, an additive response will be observed, whereas a shared binding surface will not increase the response on addition of the second domain. We performed this experiment with the injection of IT4var13^DBLβ^ followed by BC12a^DBLβ^ (Fig. 6G) and, in a separate experiment, with the injection of IT4var41^DBLβ^ followed by J1a^DBLβ^ (Fig. 6H). In each case, the injection of the second domain did not result in additional response units to that of the first domain, suggesting that the DBLβ domains tested here have an overlapping binding site.

### Binding of BC12a^DBLβ^, J1a^DBLβ^ and IT4var13^DBLβ^ to ICAM-1 mutant proteins

In an effort to better understand the differences in binding kinetics between the DBLβ domains, we used SPR to test binding of recombinant BC12a^DBLβ^, J1a^DBLβ^ and IT4var13^DBLβ^ to four ICAM-1 mutant proteins (ICAM-1^S22A^, ICAM-1^Kilifi^, ICAM-1^L42A^ and ICAM-1^L44A^) that have been shown to have differential effects on binding of different parasite isolates^25,26^. Each of the four ICAM-1 mutations obliterated binding of BC12a^DBLβ^ with only minimal signal detected (Table 3 and Fig. S3). Binding to all mutants is <8% of the full-length five domain reference ICAM-1 (ICAM-1^D1D5^) binding, although it should be noted that signals are close to background noise levels and, therefore, may not be accurate and are presented as representative values only. Similarly, J1a^DBLβ^ only minimally interacted with the ICAM-1 mutant proteins (Table 3 and Fig. S4). Binding to ICAM-1^Kilifi^, ICAM-1^L42A^ and ICAM-1^L44A^ is ≤5% of wild type ICAM-1^D1D5^ binding. ICAM-1^S22A^ binding is slightly higher at 11% of wild type binding but is still very low.

**Table 3:** The effect of ICAM-1 mutations on DBLβ domain binding.

	% ICAM-1^D1D5^ binding			
	ICAM-1^S22A^	ICAM-1^Kilifi^	ICAM-1^L42A^	ICAM-1^L44A^
BC12a^DBLβ^	2.9	7.7	6.7	6.7
J1a^DBLβ^	10.9	5.4	1.4	2.7
IT4var13^DBLβ^	94.5	8.9	3.1	10

IT4var13^DBLβ^ also had minimal interaction with ICAM-1^Kilifi^, ICAM-1^L42A^ and ICAM-1^L44A^ which is ≤10% of ICAM-1^D1D5^ binding (Table 3 and Fig. S5). However, IT4var13^DBLβ^ did bind to ICAM-1^S22A^ with 9.82 nM affinity. Therefore, the S22A mutation decreases IT4var13^DBLβ^ binding affinity for ICAM-1 but only by 5% (Table 3). This is a minor effect compared to BC12a^DBLβ^ and J1a^DBLβ^ for which binding is reduced by ≥90%, suggesting that this residue is not critical for IT4var13^DBLβ^ binding.

Overall, the mutant ICAM-1 binding assays suggest that the DBLβ domains share an overlapping but not identical binding surface in the area of the mutations. The Kilifi, L42A and L44A mutations dramatically reduce the binding ability of all three DBLβ domains. The S22A mutation has a similarly dramatic effect on BC12a^DBLβ^ and J1a^DBLβ^ binding but has minimal effect on IT4var13^DBLβ^ binding, suggesting a specific difference in contact residues in this area.

### Flow adhesion assays

The effect of the ICAM-1 mutations on recombinant DBLβ domain binding does not reflect the initial parasite binding study which assessed IE binding in static adhesion assays^26^. In light of this, we performed flow adhesion assays to test binding of BC12, J1 and IT4var13 parasite isolates to ICAM-1^D1D5^ and the four ICAM-1 mutant proteins (Fig. S6) (Data for the flow assays is shown in S1 Data). In the majority of cases, the ICAM-1 mutations resulted in a 16–56% reduction in binding of all three parasite lines, not reaching statistical significance (Fig. S6(A) and (B)). Exceptions are the L42A and L44A mutations. ICAM-1^L42A^ resulted in a 78 and 76% reduction of J1 and IT4var13 binding, respectively, and ICAM-1^L44A^ resulted in an 88% reduction of IT4var13 binding. RT-qPCR confirmed the dominantly expressed *var* gene of BC12 to be BC12a and IT4var13 to be *var13* (Fig. S6(C)). The dominant *var* of J1 is J1a which represents 62% of expressed *vars* with a number of other *var* genes making up the remaining 38% (Fig. S6). This includes the previously identified secondary tags J1d and J1b and also includes J1f which is represented at a higher level than previously observed (Fig. 1B). Thus, while there are common outcomes in binding between parasite flow adhesion assays and recombinant DBLβ domain SPR assays, such as the impact of the L42A and L44A mutations, these findings also reveal differences in results from the two formats.

## Discussion

Identifying expressed *var* genes in *Plasmodium falciparum* infected erythrocytes often starts using DBLα tag primers^15^ that target conserved homology blocks of the DBLα domain and are thought to amplify the majority of *var* genes. Indeed, such primers are frequently used in surveys of *var* gene diversity^6,27–29^. However, universal amplification by these primers cannot be known for certain due to the high variability of *var* genes. This, combined with reliance on cloning, which can carry bias towards some sequences, results in uncertainty that all expressed DBLα tags have been identified. However, in our data the most frequently cloned tag did prove to be the dominantly expressed tag in all isolates, indicating that any bias only affects secondary tags, at least in these culture-adapted isolates. The over-representation of some minor tags identified by cloning has been observed previously^30^ and highlights the importance of the more quantitative RT-qPCR method. Repeating ICAM-1 selection and RNA extraction increases confidence in this method, as was performed on BC12 and PCM7 isolates (3 replicates each). However, this proved difficult with isolate J1 (1 RNA extraction) which tended to produce gametocytes. This was also the case with several other isolates from the initial characterisation study^26^ from which sufficient RNA could not be extracted due to gametocytogenesis (data not shown).

We performed ICAM-1 selection of these isolates to identify new ICAM-1 binding *var* genes but this is not necessarily selecting for a clonal population, as multiple *var* genes can still be expressed within a selected culture. Therefore, expression of secondary tags in J1 and PCM7 can be explained by multiple *var* genes within an isolate with the ability to bind ICAM-1, as seen with multiple IT4 *var* genes^18^. Alternatively, *var* gene switching may have taken place after selection which occurs in an ordered manner and involves initially switching to numerous genes before a single dominant gene emerges^31^. We minimised the effect of switching by extracting RNA as quickly as possible after ICAM-1 selection. Whether the secondary *var* genes identified here also have the ability to bind ICAM-1 is yet to be determined.

The *var* gene database, which is a result of the Pf3k genome sequencing project, is a new resource with the potential to provide valuable information on an unprecedented scale. However, the copious amount of ‘omics’ data now being generated presents the challenge of interpretation and of identifying practical applications. We wanted to test whether this resource can predict full length *var* genes from their short ~400 bp DBLα tags. The results presented here from ICAM-1 binding patient isolates were remarkable, with the majority of successfully cloned fragments sharing ≥99% identity with the reference sequence (Fig. 2). The two cases of differential sequence (J1d_1-567 and PCM7a_4798-6762) occurred at a single point in the sequence suggesting a gene recombination event or possibly a sequence assembly error. The former is likely as it has been reported that mitotic recombination frequently occurs in *var* genes^2,32^. Interestingly, recombination occurs between domains of the same type resulting in in-frame products and domain structure preservation, which produces viable genes^2^, concordant with our results. A later search of the database containing 2512 parasite genomes found the presence of PCM7a_4798-6762 sequence and, in addition, the full length (PCM7a_1-6762) sequence (data not shown). This is perhaps unsurprising as the Pf3k project is ongoing and the database is constantly expanding. It must also be kept in mind that these genes are constantly undergoing recombination events, both mitotic^2,32^ and meiotic in the mosquito vector^33^. Therefore, although the sequences might be known, the order in which they are combined in any one gene is still unpredictable.

Limitations of the database include the varying number of hits to each DBLα tag, in particular J1a for which there were 153 hits, and the length of contiguous sequence, which varies in the database and may only provide partial sequence. In practice, there are ways to overcome some of the limitations, for example, we were able to sequence the 3′ end of *var* genes of J1b and PCM7a using a conserved exon 2 primer^16^. However, we were unable to clone and sequence exon 2 PCR products of BC12a, J1a, J1d or PCM7d and could only make size predictions of all except the PCM7d products. The un-sequenced region of J1a is the longest and is the result of having 153 returned database hits which differed significantly from the original tag sequence in the 3′ end (although several of these returned hits were identical to each other) making specific primer design impossible in this region.

Classification of *var* genes is based on a combination of upstream sequence, genomic location and direction of transcription^3,34^. Group A genes have been associated with severe disease^35–37^ as well as group B in some studies^37,38^. The majority of ICAM-1 binding *var* genes identified in the reference strains IT4 and 3D7 are group B and C type^18,19^ with only two group A ICAM-1 binders identified in 3D7: PF11_0521^39,40^ and PFD1235w^20,35^. Both UPS A ICAM-1 binding genes have been shown to induce adhesion blocking (rat) antibodies to their recombinant domains^20,40^, with PFD1235w specific antibodies additionally able to block binding of other DC4 expressing parasites, displaying cross-reactivity^20^. The dominantly expressed *var* genes of our ICAM-1 binding patient isolates are UPS B type and one UPS C type, an unsurprising result given that group A *var* genes are seldom expressed *in vitro*^19,41,42^. This is thought to be related to high switching bias towards centrally located and/or short *var* genes (4 extracellular domains), all of which are UPS B or C, rather than the specific avoidance of UPS A gene expression^43^. Assuming IT4 and 3D7 strains are representative of all parasites then we expect only 0–2 group A ICAM-1 binding *var* genes per genome and their identification from these patient isolates would require alternative methods to those employed here. For example, whole genome sequencing would identify the full *var* repertoires of these isolates and allow analysis of their predicted binding functions. However, sequence predictions alone cannot fully predict ICAM-1 binding.

The recombinogenic nature of *var* genes precludes their accurate analysis by phylogenetics. We tried to overcome this difficulty in associating sequence data with phenotype by using a similarity method that does not relate to evolutionary history, but clusters by global identity. It is interesting that the limited set of ICAM-1-binding domains do tend to cluster based on sequence identity but represent different DBLβ and UPS types (Fig. 5). However, a comparison of amino-acids from our newly sequenced DBLβ domains with those identified as important in ICAM-1 binding of UPS A, DC4-containing PfEMP1^44^ revealed very little sequence conservation (data not shown), suggesting the binding of UPS B and C *var* genes to ICAM-1 is mediated by distinct amino-acids. Further evidence of distinct UPS A binding sites is provided by blocking of binding to ICAM-1 by antibody recognising DC4 DBLβ domains in this class of variants but not being blocked by antibody recognising other DBLβ ICAM-1-binding domains (ITvar16 DBLβ5)^20^. This could point to two distinct origins of ICAM-1 binding, segregating UPS A from UPS B and C *var* genes^44^. Such segregation would be maintained by the mechanisms of *var* gene recombination which occur between domains of the same type and are mainly restricted to within UPS groups due to chromosomal location and orientation of transcription^2,3,45,46^.

We successfully expressed and purified the newly identified DBLβ domains BC12a^DBLβ^ and J1a^DBLβ^, from the dominantly expressed *var* genes of the ICAM-1 selected patient isolates BC12 and J1, respectively, to >90% purity and performed SPR to assess ICAM-1 binding, along with the previously characterised IT4var13^DBLβ^. BC12a^DBLβ^, J1a^DBLβ^ and IT4var13^DBLβ^, that all bind ICAM-1^D1D5^ with nanomolar affinity. IT4var13^DBLβ^ has the strongest interaction, followed by J1a^DBLβ^ and BC12a^DBLβ^ (K_D_ values: 3.45, 51.7, 197 nM, respectively, Fig. 6 and Table 2). These K_D_ values are similar to those found previously for five IT4 DBLβ domains, tested in comparable SPR assays, which ranged from 2.6 nM for IT4var13^DBLβ^ (comparable to our 3.45 nM value for this domain) to 144 nM for IT4var31^DBLβ ^^22^. The UPS A type, DC4 DBLβ domain was also found to bind ICAM-1 with nanomolar affinity (7.9 nM) in an SPR assay where the DBLβ domain was immobilised on the chip and ICAM-1^D1D2^ passed over^44^. All three domains studied here are DBLβ5 type and these findings fit with previous observations that, to date, all domains of this type mediate ICAM-1 binding^19^. The difference in binding kinetics between IT4var13^DBLβ^ and J1a^DBLβ^ is very interesting. They have a similar fast association rate which can be thought of as necessary to overcome the flow rate of the capillaries to mediate IE binding. Indeed, ICAM-1 has been shown to improve efficiency of IE capture under flow conditions^47^. However, they differ in their dissociation rates. IT4var13^DBLβ^ has a slow dissociation rate which fits with stationary adhesion of the IE. J1a^DBLβ^, however, has a very fast dissociation rate which could suggest that this domain could mediate rolling adhesion, in which the IE constantly attaches and detaches from the capillary wall in a rolling motion^47,48^, although care needs to be taken in extrapolating these types of data to the situation *in vivo*.

The K_D_ value is calculated based on the association and dissociation rates. In this case, the kinetic parameters fit with the response units recorded for each domain, i.e. the highest response was by IT4var13^DBLβ^, followed by J1a^DBLβ^ then BC12a^DBLβ^. The lower binding responses recorded for J1a^DBLβ^ and BC12a^DBLβ^ are in agreement with the initial parasite binding study which found both parasite isolates to be low avidity ICAM-1-binders, with BC12 having a lower binding rate than J1 (1362 and 2391 IE/mm^2^, respectively)^26^. Interestingly, the IT4var13 parasite line has a lower binding rate (900 IE/mm^2^) than both BC12 and J1 in static adhesion assays^49^, a result that is also apparent in our flow adhesion assays (Fig. S6), highlighting differential results between IEs and recombinant protein. There is no clear correlation between K_D_ values of DBLβ domains and the number of bound IEs in static adhesion assays. For example, IT4var16^DBLβ^ has the same K_D_ as J1a^DBLβ^ (51.1 and 51.7 nM, respectively) but parasites expressing *IT4var16* have more than twice the binding rate of J1 parasites in static assays (5000 and 2391 IE/mm^2^, respectively)^22,26,49^. There could be several explanations for this based on the display of host receptors on plastic and in a membrane, as well as the context of the DBL domain in its corresponding PfEMP1. The binding of an infected erythrocyte to an endothelial surface is far more complex that a simple protein-protein interaction, and many factors, including PfEMP1 architecture, PfEMP1 expression levels and knob-numbers will come into play in addition to the monovalent binding affinities measured in our SPR experiment, making a direct relationship between binding affinity and infected erythrocyte adhesion unlikely.

**Figure 7:**
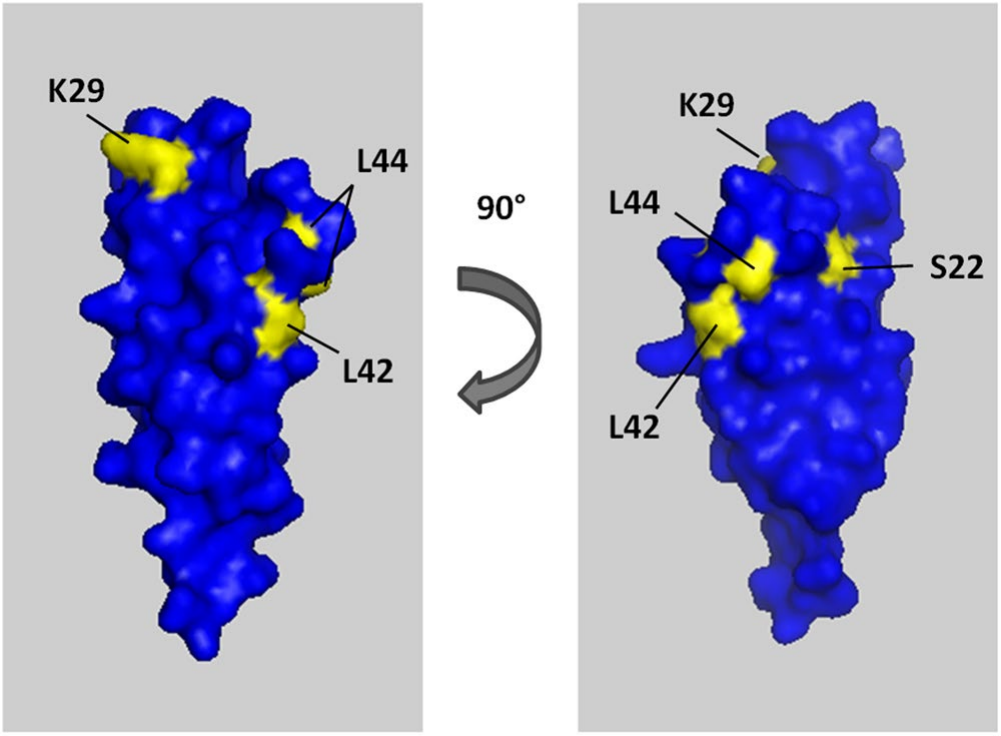
The crystal structure of ICAM-1 D1, showing the surface architecture. Residues of the mutated proteins tested in this study are labelled and coloured yellow. K29 residue is mutated to M in ICAM-1^Kilifi^. Structure accessed via Protein Data Bank, deposited by^61^.

The DBLβ binding site on ICAM-1 has been identified by mutagenesis studies and a number of ICAM-1 blocking antibodies as the BED side of D1^25,50,51^, plus the structure of the ICAM-1/DC4 (upsA) PfEMP1 complex has been solved^52^. The four ICAM-1 mutants utilized in this study have been shown to have differential effects on the binding of different parasite isolates^25,26^. In our SPR assays, the Kilifi (K29M), L42A and L44A mutations dramatically reduce the binding ability of all three recombinant DBLβ domains by ≥90%. The S22A mutation similarly reduces BC12a^DBLβ^ and J1a^DBLβ^ binding by ≥90% but has minimal effect on IT4var13^DBLβ^ binding, suggesting a specific difference in contact residues in this area. When the surface architecture of ICAM-1 is considered (Fig. 7), the S22 residue is near the edge of the area identified as important for DBLβ binding. It is likely that IT4var13^DBLβ^ has a structural difference in the area that contacts here, resulting in the lack of effect of this mutation. This has implications for development of therapeutics such as drugs or antibody therapy, which should focus around the L42 and L44 residues and possibly extend to K29 (mutated in ICAM-1^Kilifi^) to confer a cross-blocking effect.

In summary, we have shown that it is possible to reconstruct the exon 1 sequence of *var* genes from short tags by comparison with the large *var* gene database produced as part of the Pf3K project. This has the potential to open up the field by making full-length PfEMP1 sequences available for analysis in conjunction with clinical and cellular properties of the corresponding patient-derived parasite isolates.

## Materials and Methods

### Parasite culture

Laboratory-adapted patient isolates BC12, J1 and PCM7, from uncomplicated malaria patients in Thailand^53^, were collected with consent in previous clinical studies. All patient-derived material, such as white blood cells, have been removed during culture. IT4var13 is a laboratory line that binds to ICAM-1 with high affinity^22,54^. All parasites were cultured in O+ human erythrocytes at 2% haematocrit, according to standard techniques^55^ using RPMI-1640 medium (Sigma) supplemented with 37.5 mM HEPES, 7 mM D-glucose, 25 ng/ml gentamicin sulphate, 2 mM L-glutamine and 10% human serum (Haematology Department, Royal Liverpool University Hospital) at pH 7.2, in a gas mixture of 96% nitrogen, 3% carbon dioxide and 1% oxygen.

### Parasite selection on ICAM-1

50 µl Protein A Dynabeads (Invitrogen) were washed three times with 500 µl 1% BSA/PBS, using a magnet to retain the beads each time. The beads were resuspended in 1% BSA/PBS and 2.5 µg ICAM-1^D1D5^ (an Fc fusion protein of ICAM-1 domains 1–5^25^) added to a final volume of 400 µl and incubated at room temperature with 15 rpm rotation for 1 hour. The beads were then washed three times with 500 µl 1% BSA/PBS using a magnet to retain the beads and resuspended in 200 µl 1% BSA/PBS. Parasite cultures were enriched for trophozoite stage using Plasmion (Fresenius Kabi France) by standard techniques^56^. Enriched parasites were resuspended in 200 µl 1% BSA/PBS and added to the ICAM-1^D1D5^ labelled Dynabead suspension. The mixture was rotated at 15 rpm for 45 min at room temperature. Two washes were carried out with 500 µl 1% BSA/PBS to remove unbound parasites. The beads were then transferred to a new flask and cultured as described.

### RNA extraction and processing

RNA was extracted as described^57^ with minor modifications. Parasite cultures were pelleted by centrifugation at 600 × g for 5 min. The supernatant was aspirated and the pellet resuspended by tapping before addition of 10 × pellet volume of pre-warmed TRIzol® Reagent (Ambion) and incubated at 37 °C for at least 5 min. TRIzol® lysed parasites were split into 1 ml aliquots and either frozen at −80 °C or RNA extraction continued immediately. To each 1 ml aliquot, 0.2 ml chloroform was added and the tube shaken vigorously for 15 seconds, left to stand for 2–3 min and centrifuged at 1,200 × g for 30 min at 4 °C. The upper aqueous layer was removed to a new 1.5 ml tube whereupon 0.5 ml isopropanol was added and mixed by inverting the tube several times before incubation at 4 °C for at least 2 hours. The tube was mixed again by inverting several times and centrifuged at 16,200 × g for 30 min at 4 °C. The supernatant was carefully removed and the pellet washed in 0.5 ml 75% ethanol in DEPC-H_2_O (diethyl pyrocarbonate treated H_2_O, Sigma). After centrifugation at 16,200 × g for 5 min, the supernatant was removed and the pellet left to air dry at room temperature. 50 µl DEPC-H_2_O was added to the pellet before incubation at 65 °C for 10 min and then placed on ice.

1.2 µg RNA was treated with DNase I (Amplification Grade, Sigma-Aldrich) according to manufacturer’s instructions. cDNA synthesis was performed with Tetro cDNA Synthesis Kit (Bioline) following the manufacturer’s instructions. The reaction comprised 8 µl DNase treated RNA (~480 ng), 1 µl Oligo (dT)_18_ primer, dNTP at 0.5 mM final concentration, 4 µl 5 × RT Buffer, 10 U RiboSafe RNase Inhibitor, 200 U Tetro Reverse Transcriptase and DEPC-H_2_O up to 20 µl. The reaction was incubated at 45 °C for 30 min, followed by 85 °C for 5 min to stop the reaction and chilled on ice.

### Genomic DNA extraction

Genomic DNA (gDNA) was extracted from parasite cultures by saponin lysis of the RBC and the QIAamp® DNA Blood Mini Kit (Qiagen). Parasite cultures were centrifuged at 600 × g for 5 min and the resulting pellet was resuspended in 5 ml 0.15% saponin and incubated for 5 min on ice, mixing each minute. Parasites were centrifuged at 600 × g for 5 min and washed with PBS 2–3 times. Following the final centrifugation, the parasite pellet was resuspended in 200 µl PBS and transferred to a 1.5 ml tube containing 20 µl Qiagen protease. The QIAamp® DNA Blood Mini Kit (Qiagen) spin protocol was followed with a final elution of gDNA in 100 µl Buffer AE (10 mM Tris- HCl, 0.5 mM EDTA, pH 9).

### Primer design and polymerase chain reaction (PCR)

All novel primer sequences were designed with the aid of OligoCalc^58^. Primer sequences from previously published work are reproduced with the appropriate citation. All primer sequences are listed in Supplementary Table S1 along with annealing temperature.

All PCR reactions were carried out with the proofreading *TaKaRa LA Taq*® DNA polymerase (Clontech, TaKaRa Bio Inc) according to the manufacturer’s instructions. Each reaction comprised 10 × LA PCR Buffer II, magnesium chloride (MgCl_2_) at 2.5 mM final concentration, dNTP at 1 mM final concentration, primers at 0.3 mM final concentration each, 2.5 units of *TaKaRa LA Taq*®, 2 µl template and sterile water up to 50 µl final volume. The DBLα tag was amplified from parasite cDNA using the primers DBLαAF′ and DBLαBR^15^. Reaction conditions were an initial denaturing step of 95 °C for 3 min, followed by 30 cycles of 95 °C for 30 sec, 47 °C for 30 sec, 65 °C for 30 sec, and a final extension of 65 °C for 3 min. 5′ UPS PCR was carried out using primers unique to the three main UPS types A, B and C^6^ in combination with a reverse DBLα specific primer designed for RT-qPCR (see Table S1). Reaction conditions were as follows: 95 °C for 3 min, followed by 30 cycles of 95 °C for 30 sec, 52 °C for 30 sec, 65 °C for 30 sec, and a final extension of 65 °C for 3 min. Downstream PCR was carried out using a primer designed to the more conserved exon 2^16^ in combination with an isolate specific forward primer (see Table S1). Reaction conditions: 96 °C for 1 minute, followed by 40 cycles of 98 °C for 6 seconds, 48 °C for 15 seconds, 67 °C for 5 minutes, and a final extension of 68 °C for 10 minutes. PCR of overlapping *var* gene fragments was carried out using primers listed in Table S1 with annealing temperatures as indication and reaction conditions of 95 °C for 3 min, followed by 30 cycles of 95 °C for 30 sec, 52–55 °C for 30 sec, 65 °C for 1 min, and a final extension of 65 °C for 3 min.

### Cloning, plasmid preparation and sequencing

PCR products were cloned into pCR^TM^4-TOPO® vector (Invitrogen) at 10:1 insert to vector molar ratio according to manufacturer’s instructions. Plasmids were transformed into One Shot® TOP10 *E. coli* competent cells (Invitrogen), grown overnight and the plasmids isolated using standard methods. Plasmid sequencing was carried out by either the Core Genomic Facility, University of Sheffield or Source Bioscience Sequencing, Rochdale UK. Sequences were visualised with Chromas Lite version 2.1 (Technelysium Pty Ltd, latest version available at http://technelysium.com.au/) and alignments were carried out using ClustalX 2.1^59^ on default settings.

### Reverse transcriptase quantitative PCR (RT-qPCR)

RT-qPCR of cDNA was carried out using Brilliant III Ultra-Fast SYBR® Green QPCR Master Mix (Agilent Technologies). Standard curves were generated for each new primer set by performing 10-fold serial dilutions of cDNA to produce five different concentrations of starting material. No-RT and DEPC-H_2_O reactions were carried out as negative controls. Each reaction comprised 10 µl 2 × SYBR Green QPCR master mix, forward and reverse primers each at 0.5 µM final concentration (see Table S1 for primer sequences), 2 µl template (cDNA, no-RT or DEPC-H_2_O) and DEPC-H_2_O to 20 µl final volume. Adenylosuccinate lyase (ASL) was used as an internal control gene^60^. Reactions were run on an MxPro-Mx3005P machine under the following conditions: 95 °C for 3 min, 40 cycles of 95 °C for 10 seconds, 60 °C for 10 seconds, and a final cycle of 95 °C for 1 min, 55 °C for 30 seconds, 95 °C for 30 seconds.

Primer efficiency was calculated from the gradient of the standard curve using the equation E = 10^1/−m^, where E is efficiency and m is the gradient of the curve. RT-qPCR was repeated on cDNA at a single concentration with the same reagents and conditions (as above) using all primer pairs designed to particular isolates on the same plate. Ct values were normalised against the internal control gene using the equation 2^−ΔCt^, where ΔCt is the mean Ct value of the gene of interest minus the mean Ct value of the internal control gene. 2^−ΔCt^ values were transformed into percentage of the total for each parasite isolate.

### Pf3k var gene database search, primer design and sequencing

Pf3k is a project set up as part of MalariaGEN which aims to sequence 3,000 *Plasmodium falciparum* genomes and is led by the University of Oxford, the Wellcome Trust Sanger Institute and the Broad Institute (https://www.malariagen.net/projects/parasite/pf3k). As a result, the *var* genes from over 2,500 clinical isolates are assessable (http://www.sanger.ac.uk/cgi-bin/blast/submitblast/p_falciparum-pf3k). The short DBLα tags generated in this study were BLAST searched against the database containing 1468 parasite genomes with parameters of at least 99% identity and 95% overlap. The returned sequence hits were used as reference genes to design primers (see Table S1) with overlapping products of ~1 kb along the length of the gene. The primers were tested on parasite gDNA (see above for PCR conditions) and the products cloned into pCR^TM^4-TOPO® vector (Invitrogen) and sequenced as described. Overlapping sequences were assembled to reconstruct each *var* gene.

### Sequence analysis

Reconstructed *var* gene sequences were translated to amino acid sequence and entered into the VarDom 1.0 Server^4^ (available at http://www.cbs.dtu.dk/services/VarDom/) in FASTA format to define domain boundaries. The corresponding nucleic acid sequences were then separated into domains and BLAST searched against the entire 7 genomes dataset^4^. The top six hits were analysed and domain subtypes were identified. Subtypes were included if a consensus between the six hits was reached or if the top hits had appropriate coverage. Where multiple subtypes were identified, they were included if there were two of equal standing (separated by “/”) or excluded if there were more than two matches with similar coverage.

Similarity matrixes were generated as follows. First domains were compared all-against-all with BLASTp, E value 1-e6. The identity of the first hit was normalised by the mean of the two compared domains, resulting in a matrix of identity between all domains, ranging from 0–100. This matrix was visualized in R using the heatmap.2 function from the gplots library. To cluster the domains, the “distance method” from the heatmap.2 function was used.

All previously described DBLβ sequences were downloaded from the VarDom 1.0 Server^4^.

### Recombinant protein expression and purification

DBLβ domains of BC12a and J1a were PCR amplified using primers shown in Table S1, subcloned into a modified pET15b vector and expressed as an N-terminal, hexahistidine tagged protein in *Escherichia coli* SHuffle 3030 cells (New England Biolabs) at 25 °C for 16 hours. Cells were pelleted and lysed, and the DBLβ domains purified by affinity chromatography using nickel-nitrilotriacetic acid agarose (Ni-NTA, Qiagen) under native conditions. DBLβ domains were further purified by gel filtration on a HiLoad 16/600 Superdex 75 prep grade column (GE healthcare).

IT4var13^DBLβ^, IT4var41^DBLβ^^22^, ICAM-1^D1D5^^44^, ICAM-1^Kilifi^, ICAM-1^S22A^, ICAM-1^L42A^, ICAM-1^L44A^^25^ were expressed and purified as described previously.

### CD spectroscopy

Far-UV CD spectroscopy experiments were carried out in a J-815 Spectropolarimeter (Jasco) equipped with a computer-controlled Peltier temperature control unit. Measurements were carried out in 100 mM Na-phosphate buffer, 200 mM NaF, pH 7.2 at a protein concentration of 0.3 mg/ml using a 1 mM path cell. All measurements were taken at 20 °C between 195 and 260 nm wavelengths. Four spectra were recorded for each sample, averaged and corrected for buffer absorption.

### Surface plasmon resonance (SPR)

SPR was carried out on a Biacore T200 machine (GE Healthcare). Protein A was immobilised onto a CM5 chip (GE Healthcare) by amine coupling. All experiments were carried out in buffer containing 10 mM HEPES, pH 7.2, 250 mM NaCl and 0.05% Tween- 20, filter sterilised and degassed. ICAM-1 proteins fused to the Fc region of human IgG1 (ICAM-1^D1D5^^44^, ICAM-1^Kilifi^, ICAM-1^S22A^, ICAM-1^L42A^, ICAM-1^L44A^^25^) were captured onto the Protein A surface. Coupling of the different ICAM-1 proteins to the chip was kept comparable at 1108 ± 108 response units (RU). DBLβ domains were flowed over the chip as a twofold dilution series (1000 nM–7.81 nM) at a flow rate of 30 µl/min with an association time of 240 seconds and a dissociation time of 600 seconds. The chip was regenerated after each concentration with the injection of 10 mM Glycine- HCl, pH 1.7 for 120 seconds at a flow rate of 10 µl/min which breaks the Protein A- Fc interaction. The signal from a flow channel without ICAM-1 was subtracted from all measurements. Single stranded DNA (ssDNA, Salmon Sperm DNA sodium salt, AppliChem) was added to BC12a^DBLβ^ to 1 mg/ml final concentration prior to injection to minimise background binding observed during test runs. Injection of ssDNA only had no effect on the signal (data not shown). Sensorgrams were fitted to a global 1:1 interaction model allowing calculation of kinetic values k_a_, k_d_ and K_D_ using BIAevaluation software 2.0.3 (GE Healthcare).

SPR competition assays were carried out with ICAM-1^D1D5^ under the same conditions outlined above except that only one concentration of DBLβ was used (adjusted to give the same molar ratio of each protein). The first domain was injected at a flow rate of 30 µl/min with an association time of 240 seconds. The second domain was then injected over the same chip at a flow rate of 30 µl/min with an association time of 240 seconds and a dissociation time of 600 seconds.

### Flow adhesion assay

Flow adhesion assays were carried out with the VenaFlux semi-automated microfluidic system and VenaFlux software (Cellix). The Vena8 Fluoro + ^TM^ protein biochips (Cellix) used contain 8 channels which allow several proteins to be tested in immediate succession. Individual biochip channels were coated with 4 µl of 50 µg/ml of either ICAM-1^D1D5^, ICAM-1^S22A^, ICAM-1^Kilifi^, ICAM-1^L42A^ or ICAM-1^L44A^ protein and incubated at 37 °C for 1 hour in a humidified petri dish. The protein was then aspirated and the channel blocked with 1% BSA/PBS for either 1 hour at 37 °C or overnight at 4 °C. Channels were warmed to 37 °C before use. Infected erythrocytes (IE) were prepared in binding buffer (RMPI 1640 powder (Invitrogen) dissolved in H_2_O, pH 7.2) at 3% parasitaemia and 2% haematocrit. The assay followed the Cellix protocol. The protein coated biochip was attached to a microscope stage within a plastic chamber whose temperature was 37 °C. The IE were drawn through the channel at a flow rate of 0.04 Pa for 8 min before washing with binding buffer. Bound parasites were counted in 7–10 microscope fields and the numbers adjusted to IE/mm^2^.

All methods were carried out in accordance with relevant guidelines and regulations. No patient material was used in this study. Parasite isolates used in this work were laboratory adapted and their derivation, including ethical clearance, is covered in the references cited. Human red blood cells for parasite culture were obtained commercially from the UK Blood Transfusion Service (NHSBT) and used under licence from the UK Human Tissue Authority.

### Data availability

All data generated or analysed during this study are included in this published article (and its Supplementary Information files) or are publicly available at www.malariagen.net/data/pf3k-5.

## Electronic supplementary material

Supplementary information

Supplementary data file

Supplementary data file

Supplementary data file

Supplementary data file

Supplementary data file

Supplementary data file

Supplementary data file
