# Supplementary material for: In silico guided reconstruction and analysis of ICAM-1-binding var genes from Plasmodium falciparum
Source: Sci Rep. 2018 Feb 19;8:3282. doi: 10.1038/s41598-018-21591-8 (PMC5818487; doi:10.1038/s41598-018-21591-8)
Supplement: Supplementary file 1 — Supplementary information [file 41598_2018_21591_MOESM1_ESM.pdf]

In silico guided reconstruction and analysis of ICAM-1-binding *var* genes from *Plasmodium falciparum*.

Eilidh Carrington[1, #a], Thomas D. Otto[2, #b], Tadge Szestak[1], Frank Lennartz[3], Matthew K. Higgins[3], Chris I. Newbold[2,4], Alister G. Craig [*,1]

[1] Liverpool School of Tropical Medicine, Pembroke Place, Liverpool L3 5QA, UK

[2] Wellcome Trust Sanger Institute, Wellcome Genome Campus, Hinxton, Cambridge CB10 1SA, UK

[3] Department of Biochemistry, University of Oxford, South Parks Road, Oxford OX1 3QU, UK

[4] Weatherall Institute of Molecular Medicine, University of Oxford, John Radcliffe Hospital, Headington, Oxford OX3 9DS, UK

[#a] Current address: Malaria Gene Regulation Lab, Swiss Tropical and Public Health Institute, Socinstrasse 57, 4051 Basel, Switzerland

[#b] Current address: Institute of Infection, Immunity and Inflammation, University of Glasgow, College of Medical, Veterinary and Life Sciences, Sir Graeme Davies Building, 120 University Place, Glasgow G12 8TA, UK

* Corresponding author

Email: alister.craig@lstmed.ac.uk

Supplementary Information

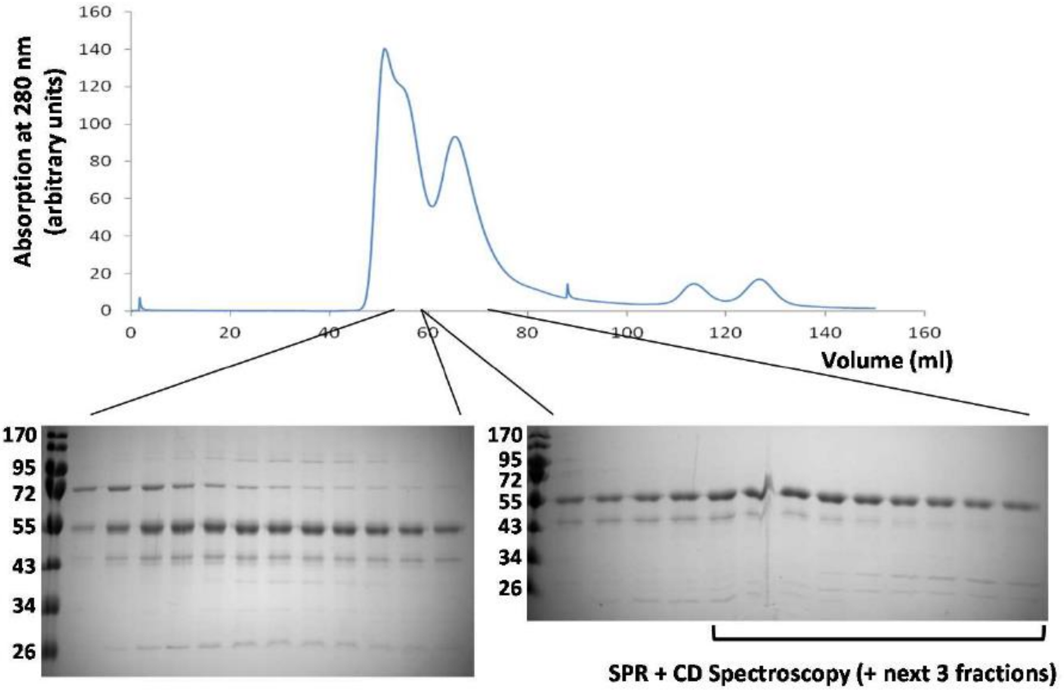

S1 Fig. Purification of BC12aDBLβ by gel filtration.

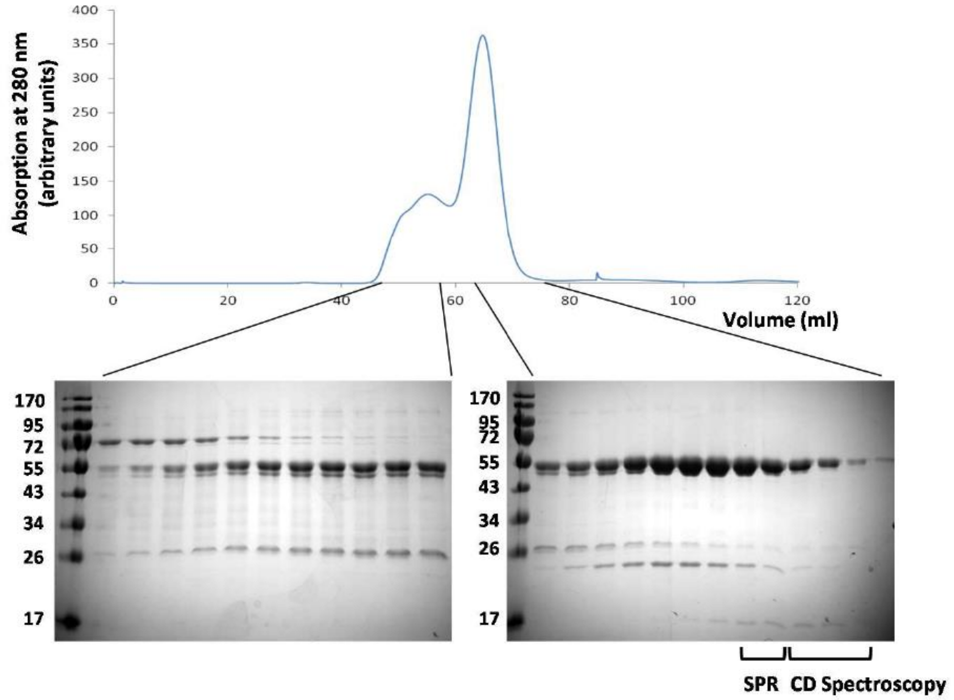

S2 Fig. Purification of J1aDBLβ by gel filtration.

For S1 Fig and S2 Fig, DBLβ domains were passed through a HiLoad 16/600 Superdex 75 prep grade column (GE healthcare) and 1 ml fractions collected between 38 and 100 ml. For the BC12DBLβ gel filtration, SDS-PAGE of samples is from fractions 11-22 (left) and 23-35 (right). Fractions pooled for

use in SPR and CD spectroscopy are indicated (black bar). For J1aDBLβ gel filtration, SDS-PAGE of samples is from fractions 11-21 (left) and 22-34 (right). Fractions pooled for use in SPR and CD spectroscopy are indicated (black bars).

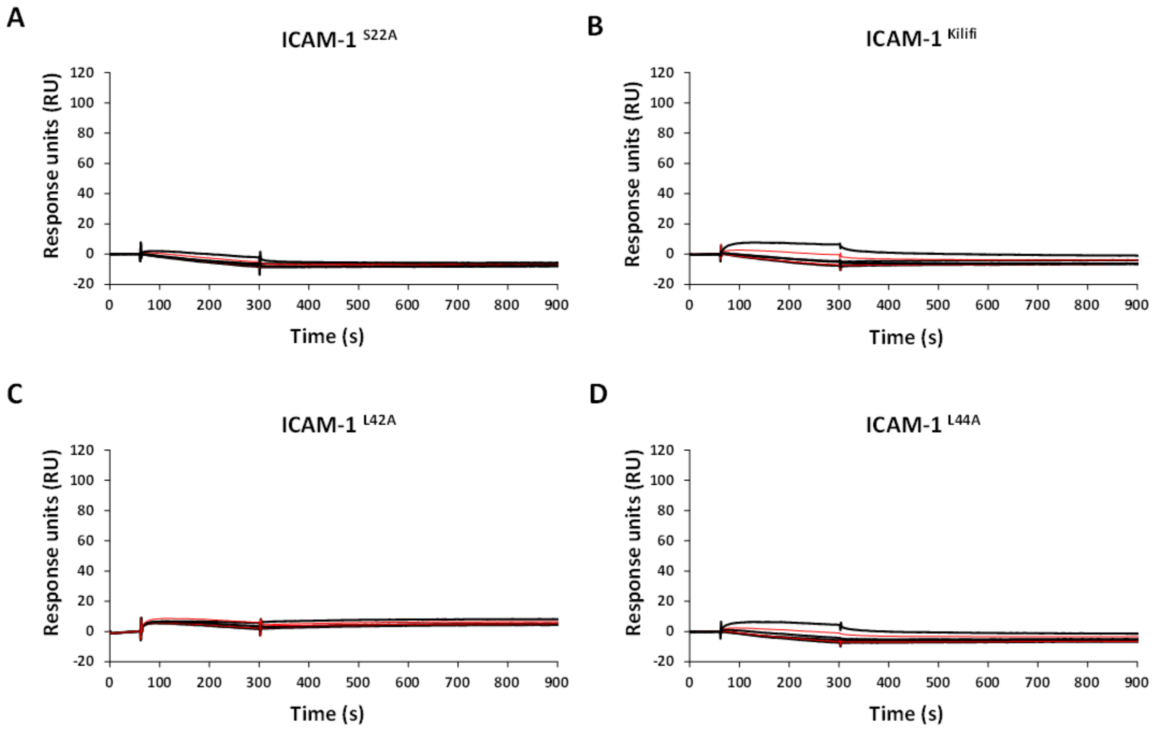

S3 Fig. BC12a$^{DBL\beta}$ response to ICAM-1 mutant proteins.

ICAM-1 mutant proteins were coupled to a sensor chip surface (1000 RU) and BC12a$^{DBL\beta}$ was injected at 30 µl/min with an association time of 240 seconds and a dissociation time of 600 seconds. Shown are sensorgrams for the binding of BC12a$^{DBL\beta}$ to ICAM-1^{S22A} (A), ICAM-1^{Kilifi} (B), ICAM-1^{L42A} (C) and ICAM-1^{L44A} (D). Data (black lines) are modelled to a 1:1 global interaction model (red lines). Data shown in S5 Data.

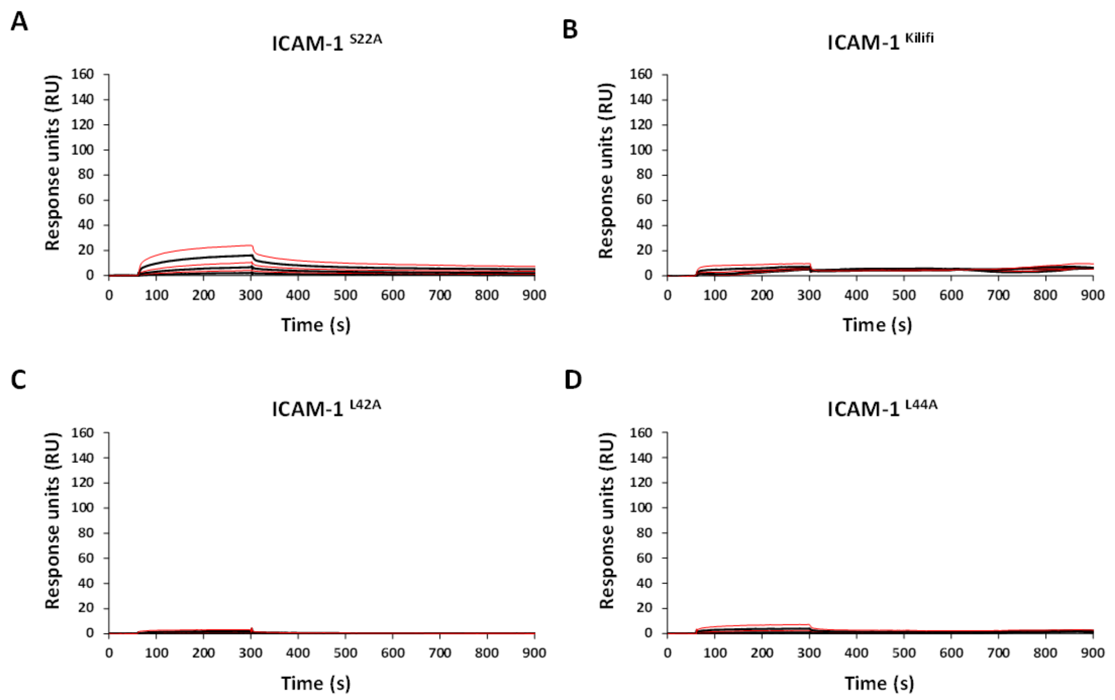

S4 Fig. J1a$^{DBL\beta}$ response to ICAM-1 mutant proteins.

ICAM-1 mutant proteins were coupled to a sensor chip surface (1000 RU) and J1a$^{DBL\beta}$ was injected at 30 µl/min with an association time of 240 seconds and a dissociation time of 600 seconds. Shown are sensorgrams for the binding of J1a$^{DBL\beta}$ to ICAM-1^{S22A} (A), ICAM-1^{Kilifi} (B), ICAM-1^{L42A} (C) and ICAM-1^{L44A} (D). Data (black lines) are modelled to a 1:1 global interaction model (red lines). Data shown in S6 Data.

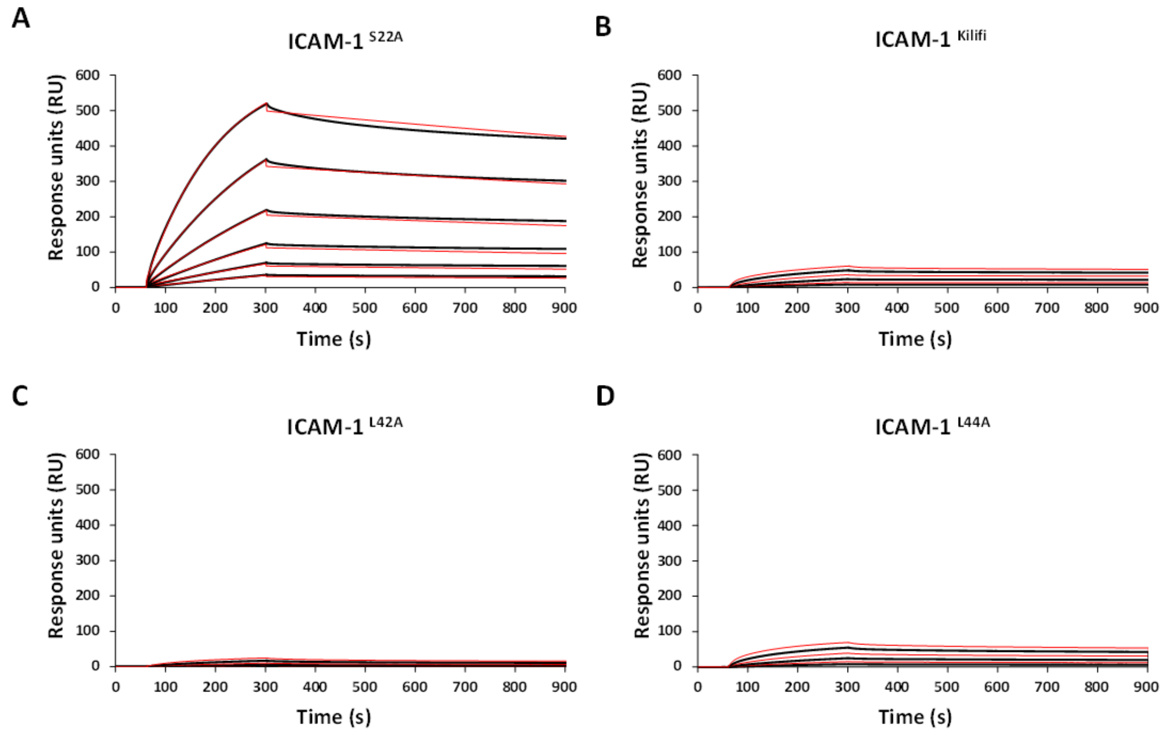

S5 Fig. IT4var13$^{DBL\beta}$ response to ICAM-1 mutant proteins.

ICAM-1 mutant proteins were coupled to a sensor chip surface (1000 RU) and IT4var13$^{DBL\beta}$ was injected at 30 µl/min with an association time of 240 seconds and a dissociation time of 600 seconds. Shown are sensorgrams for the binding of IT4var13$^{DBL\beta}$ to ICAM-1^{S22A} (A), ICAM-1^{Kilifi} (B), ICAM-1^{L42A} (C) and ICAM-1^{L44A} (D). Data (black lines) are modelled to a 1:1 global interaction model (red lines). Data shown in S7 Data.

A

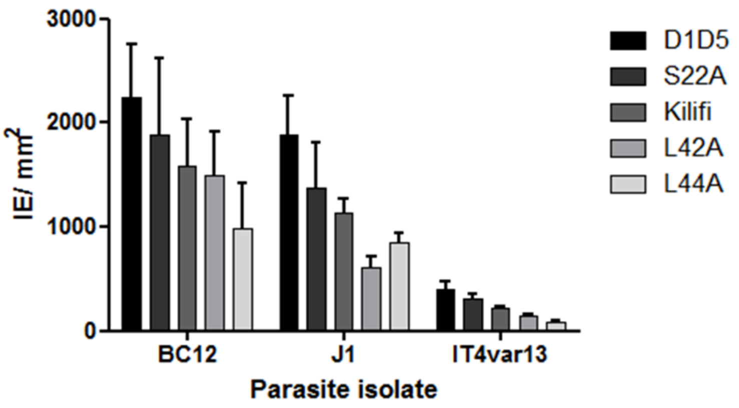

B

	ICAM-1^{S22A}	ICAM-1^{Kilifi}	ICAM-1^{L42A}	ICAM-1^{L44A}
**BC12 (n=3)**	0.708	0.387	0.318	0.134
**J1 (n=3)**	0.428	0.139	0.033	0.059
**IT4var13 (n=4)**	0.325	0.061	0.016	0.007

C

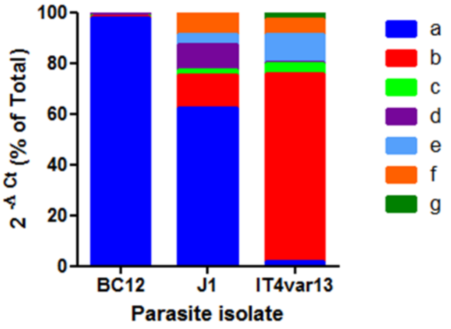

S6 Fig. Adhesion of BC12, J1 and IT4var13 parasite isolates to ICAM-1 proteins under flow conditions.

(A): Individual biochip channels were coated with 4 µl of 50 µg/ml of either ICAM-1^{D1D5}, ICAM-1^{S22A}, ICAM-1^{Kilifi}, ICAM-1^{L42A} or ICAM-1^{L44A}. Recently ICAM-1-selected IEs were passed through the channel at 3% parasitaemia, 2% haematocrit for 8 minutes. Bound parasites were counted in 7-10 fields and adjusted to IE/mm^2. Bars represent the mean of 3 (BC12, J1) or 4 (IT4var13) independent experiments with the standard error of the mean (SEM) shown. Binding data shown in S1 Data. (B):

P-values as a result of unpaired t-tests comparing each ICAM-1 mutant protein to the wild type ICAM-1^{D1D5}. (C): RT-qPCR of cDNA isolated from each parasite culture. RT-qPCR was carried out using BC12 and J1 DBLα tag primers (a-e and a-f, respectively) and primers to IT4 ICAM-1 binding *var* genes (a: 01, b:13, c:14, d:16, e:27, f:31, g:41). Primers are listed in Table S1. Ct values were normalised against the ASL internal control gene to give $2^{-\Delta Ct}$ values and are shown as percentage of total for each isolate.

Table S1. Primer sequences

DBLα tag, UPS and Exon 2 previously published primers

Target	T_A (°C)	Primer name	Sequence 5'-3'
**DBLα tag †**	47	DBLαAF'	GCACG(A/C)AGTTT(C*/T)GC
**DBLα tag †**	47	DBLαBR	GCCCATTC(G/C)TCGAACCA
**UPS A ‡**	52	upsA-5'	ATTAYATTTGTTGTAGGTGA
**UPS B ‡**	52	17DBLa-5'	ATGTAATTGTTGTTTTTTTTTTTGTTAGAATATTTAAA
**UPS C ‡**	52	5B1-5'	CACATATARTACGACTAAGAAACA
**Exon 2 ˢ**	48	Ex2-reg	TCTTCATAYTCRCTTTC

† (Bull *et al.*, 2005), ‡ (Mugasa *et al.*, 2012), ˢ (Lavstsen *et al.*, 2012)

ICAM-1 binding isolate RT-qPCR primers

Target	T_A (°C)	Primer name	Sequence 5'-3'
**DBLα tag BC12a**	60	E-195F	AAGCGGAAAAACACTACGAAGAT
**DBLα tag BC12a**	60	E-196R	TAGCCATAGATGGACTTTCACTA
**DBLα tag BC12b**	60	E-197F	AAGCGCAACACAAAAGCATTATGCA
**DBLα tag BC12b**	60	E-198R	TTTGTTATATGTTATATTTCCTCCATTACA
**DBLα tag BC12d**	60	E-201F	GACGAGAGGGAGGACGAA
**DBLα tag BC12d**	60	E-202R	ATTATCACCACCACATGTTTTTCGA
**DBLα tag BC12e**	60	E-203F	AGATCTCGCTACAAAAAAGACGGT
**DBLα tag BC12e**	60	E-204R	TAGCAGTCGTACCGTATGTGAT
**DBLα tag BC12f**	60	E-205F	ACGGATCCCAAAGCGAAAGA
**DBLα tag BC12f**	60	E-206R	TTGGGCTCCACTTTGTCCAT
**DBLα tag J1a**	60	E-183F	TCGTACTACAAAAATGATAATGACCG
**DBLα tag J1a**	60	E-184R	ATGCCCACCCTTAATGGAAGA
**DBLα tag J1b**	60	E-185F	AGACGAATGGGAAGGTACCAGA

**DBLα tag J1b**	60	E-186R	AGCCGAACTTCCTCCTTCAGA
**DBLα tag J1c**	60	E-211F	AGGCAGCRAAAGACCACTAC
**DBLα tag J1c**	60	E-212R	TTCTCCTGAACCACATGTATTTCTA
**DBLα tag J1d**	60	E-213F	AAGAAGAACAATAAGAATCCGGCAA
**DBLα tag J1d**	60	E-214R	TCTAAAATATTCAGCATTATCTGTTACA
**DBLα tag J1e**	60	E-215F	AGACGAATGTGAAGACGAATGT
**DBLα tag J1e**	60	E-216R	TGCACACGCTCTTTGTCGAA
**DBLα tag J1f**	60	E-217F	AGAGGGAAGAAAGGCGCAA
**DBLα tag J1f**	60	E-218R	AGAGTCATACCATCATCTGCGAT
**DBLα tag PCM7a**	60	αE-3F	TGGATTGATGAACGGCGCACA
**DBLα tag PCM7a**	60	αE-4R	TACCACTCTTAACGTCACACG
**DBLα tag PCM7b**	60	αE-1F	AGATCGCTACCAAGATACTGA
**DBLα tag PCM7b**	60	αE-2R	CATTCCAAAGAGTCATACCAT
**DBLα tag PCM7c**	60	E-269F	AAGATGAACAAATTAGGGAATACTGGT
**DBLα tag PCM7c**	60	E-270R	TGGCATTTATCCAGACTCCAAGT
**DBLα tag PCM7d**	60	E-271F	ACAATGATGATACTGACAAAAACTATTACA
**DBLα tag PCM7d**	60	E-272R	AGGCTCTCCATTGACACATCTA
**DBLα tag PCM7e**	60	E-273F	TACAAACTCGCTACGGAAGTGAT
**DBLα tag PCM7e**	60	E-274R	AGTTCCATCGATACACTGGCA

IT4 RT-qPCR primers (Viebig *et al.*, 2007)

**Target**	**T_A (°C)**	**Primer name**	**Sequence 5'-3'**
**IT4var01**	60	IT4var01F	GATCCGCCAGCAAAAGAAG
**IT4var01**	60	IT4var01R	CCCCCTTTATATTTTTGTCTGC
**IT4var13**	60	IT4var13F	GTAAACATCAGGCGTGTAAGG
**IT4var13**	60	IT4var13R	TGTTCCTCTCCGCTGAAGA
**IT4var14**	60	IT4var14F	CAAGATGGAAGCGGTAAAG
**IT4var14**	60	IT4var14R	CATGCATTATCCCAAAGAT
**IT4var16**	60	IT4var16F	ATGGTAGACAAGCTGTTCGTTT
**IT4var16**	60	IT4var16R	AGCACAGGCTCCTACTGAATT
**IT4var27**	60	IT4var27F	CAATAACGACAACCCTGGCA
**IT4var27**	60	IT4var27R	TGGTGTCTTCGTCGGTTTTT
**IT4var31**	60	IT4var31F	ACTGGTCGTAAAGGTGCACA
**IT4var31**	60	IT4var31R	CTCCCTTCAAATCACTTCCC
**IT4var41**	60	IT4var41F	AACATATGTTTGATAGAATTG
**IT4var41**	60	IT4var41R	TGGCATCTGTAGGCACGAA

Primers designed against *var* database hits to ICAM-1 binding isolates

Target	T_A (°C)	Primer name	Sequence 5'-3'
**XX0156-C.g40 (BC12a)**	52	E-227F	TAGTGAAAGTCCATCTATGGCTA
**XX0156-C.g40 (BC12a)**	52	E-228R	TCCATTCATCATACAGTTTCTGACT
**XX0156-C.g40 (BC12a)**	52	E-229F	TGGTGATGGACAAACAGAAATTGAA
**XX0156-C.g40 (BC12a)**	52	E-230R	ACGCCTTTCTACCACCAGCA
**XX0156-C.g40 (BC12a)**	52	E-231F	ACCTATTATGAGATCCAATCCATGT
**XX0156-C.g40 (BC12a)**	52	E-232R	TTTCTCATATTTATCGCAGGCGTTT
**XX0156-C.g40 (BC12a)**	52	E-233F	TGCAATCACAGGAGTATGAGACA
**XX0156-C.g40 (BC12a)**	52	E-234R	TTCTTTAGGTTTTGGGAATATTGTATCT
**XX0156-C.g40 (BC12a)**	52	E-235F	AGGTCCCTCCAGAATTTTTGC
**XX0156-C.g40 (BC12a)**	52	E-236R	TGGGTGGCACACAAATACTAC
**XX0156-C.g40 (BC12a)**	52	E-237F	AGTCTCAATGCCGCCTGCT
**XX0156-C.g40 (BC12a)**	52	E-238R	TTGACCCCATTCTTCAAGGTAAC
**XX0156-C.g40 (BC12a)**	52	E-239F	TGTCACACTTAAAGATGAAAGTAGT
**XX0156-C.g40 (BC12a)**	52	E-240R	ATGGGGATTCTTCACGATTTTTCATA
**XX0156-C.g40 (BC12a)**	52	E-241F	ATGAGGAAAAAGTCAGTGGGAAA
**XX0156-C.g40 (BC12a)**	52	E-242R	AGTGAACGCAGCAAAACCGAT
**XX0156-C.g40 (BC12a)**	52	E-242F	ATCGGTTTTGCTGCGTTCACT
**XX0004-C.g20 (J1a)**	52	E-257F	TCTTCCATTAAGGGTGGGCAT
**XX0004-C.g20 (J1a)**	52	E-258R	ACATTTACTAATTTCTTTCCTCCATTCT
**XX0004-C.g20 (J1a)**	52	E-259F	TGATGAAGATAATGACTGTGAAACGA
**XX0004-C.g20 (J1a)**	52	E-260R	ACATATGTTCTCTTCTTTGTGGCA
**XX0004-C.g20 (J1a)**	52	E-261F	TGAAAATCATTCCAATCGTAATCCTAA
**XX0004-C.g20 (J1a)**	52	E-262R	ACTATTTGGAGGGAGTAATTCTTGTA
**XX0004-C.g20 (J1a)**	52	E-263F	ATGGGACAAAATGCAACTGAAATACT
**XX0004-C.g20 (J1a)**	52	E-264R	ATTATCGCCACTAAATGTAACGGTTT
**XX0137-C.g35 (J1a)**	52	E-309F	AAAGGTGACCAAACAGATGAAAAGAA
**XX0137-C.g35 (J1a)**	52	E-310R	AGTGATAAATATCTATCTCCATCTGTTGT
**XX0137-C.g42 (J1b)**	52	E-243F	ATGCCAACTTAATCACTCTCTTCAT
**XX0137-C.g42 (J1b)**	52	E-243R	ATGAAGAGAGTGATTAAGTTGGCAT
**XX0137-C.g42 (J1b)**	52	E-244R	TGTTTCAGCACAGAACGCGTT
**XX0137-C.g42 (J1b)**	52	E-245F	AGTGGTACTAATGATAAAGAAAAAGGAA
**XX0137-C.g42 (J1b)**	52	E-246R	TGTGGGTCTGGGCACTTGT
**XX0137-C.g42 (J1b)**	52	E-247F	AAGAAACAAAACGCATTAAGGACAT
**XX0137-C.g42 (J1b)**	52	E-248R	TGTGGGAGGAGGTTTGGAA
**XX0137-C.g42 (J1b)**	52	E-249F	ATATAGTAGTACGTGGTGTTGCT
**XX0137-C.g42 (J1b)**	52	E-250R	AGTTTTGCAGTCCTTTTCTTTACCAT
**XX0137-C.g42 (J1b)**	52	E-251F	AGGTAGTGAGATAACTTTTGATGATA
**XX0137-C.g42 (J1b)**	52	E-252R	TTCATCACTATTATCTGCTGGTTCA
**XX0137-C.g42 (J1b)**	52	E-253F	TGAACCAGCAGATAATAGTGATGAA

**XX0382-C.g38 (J1d)**	52	E-254R	TGTAACCTCATAATAAGAACATTGCCA
**XX0382-C.g38 (J1d)**	52	E-255F	AGTAATAAAGAGCGTGAGAATAATCCT
**XX0382-C.g38 (J1d)**	52	E-256R	ACCTCACTTGACGGCCATCT
**XX0352-C.g23 (J1d)**	52	E-275F	ACAGAAAAGGGGGTAAAGCAGAT
**XX0352-C.g23 (J1d)**	52	E-276R	TCAATCGCATGCTTGTCTTTATCTTT
**XX0352-C.g23 (J1d)**	52	E-277F	AAAATCAATGGAAACAAATGGACGAA
**XX0352-C.g23 (J1d)**	52	E-278R	TGTGATAGACCACATAACATTCCT
**XX0352-C.g23 (J1d)**	52	E-279F	AGGTAGTGATGTGAGTGATGTAGA
**XX0352-C.g23 (J1d)**	52	E-280R	ACCTGTTTTCAAACGATTCATACCT
**XX0352-C.g23 (J1d)**	52	E-281F	AAGCATACATGAAGGATCAGAAGATT
**XX0352-C.g23 (J1d)**	52	E-282R	CTGTCGCGGCCTTGCATTTA
**XX0352-C.g23 (J1d)**	52	E-283F	TAAAGAATACAGCATACTTGTTAGTAA
**XX0352-C.g23 (J1d)**	52	E-284R	TCTGTAAAGTGTTTCTCACCCATA
**VAR0141-C.g21 (PCM7a)**	55	E-15F	TGTGGTGGAGGAAATCTAAC
**VAR0141-C.g21 (PCM7a)**	55	E-16R	CTTCTCCGCAGGTTTCGTGACC
**VAR0141-C.g21 (PCM7a)**	55	E-17F	CATATTGCGAAGCATGTCCGTG
**VAR0141-C.g21 (PCM7a)**	55	E-18R	GCATCCACATGTGTATCTTC
**VAR0141-C.g21 (PCM7a)**	55	E-19F	GGCAACACATGTATAACAGG
**VAR0141-C.g21 (PCM7a)**	55	E-20R	GGTGGCATGTAGATATCTTCGGC
**VAR0141-C.g21 (PCM7a)**	55	E-21F	GCAAGATTGACACAACGTATTCC
**VAR0141-C.g21 (PCM7a)**	55	E-22R	CTTACACGTACCACTAACACCCG
**VAR0141-C.g21 (PCM7a)**	55	E-23F	GTCCTGGTATGCCAGTTGACG
**VAR0141-C.g21 (PCM7a)**	55	E-24R	GCCATATTTGAGGTTGCATGC
**VAR0141-C.g21 (PCM7a)**	55	E-25F	GAGGAGGAAGTCACTGACGAC
**VAR0141-C.g21 (PCM7a)**	55	E-26R	CACCAGTCAACACGCTTGTCAC
**VAR0141-C.g21 (PCM7a)**	55	E-27F	GATTAAGGCGTTAGAAGCTAGTGG
**VAR0141-C.g21 (PCM7a)**	55	E-28R	GCAGCGTCAGTGCACGTATCTAG
**VAR0141-C.g21 (PCM7a)**	55	E-29F	GTGCCATTTCTTGTAGATC
**VAR0141-C.g21 (PCM7a)**	55	E-30R	CAGTTCTCTGTTCGAATTGCTC
**VAR0141-C.g21 (PCM7a)**	55	E-31F	GGCCATGTATAGAAAATGG
**VAR0141-C.g21 (PCM7a)**	55	E-32R	GGAAGCGGACATAACATCGC
**XX0488-C.g40 (PCM7d)**	52	E-289F	AACCCCTTTCAGAACTTTGTCAT
**XX0488-C.g40 (PCM7d)**	52	E-289R	ATGACAAAGTTCTGAAAGGGGTT
**XX0488-C.g40 (PCM7d)**	52	E-290R	TACACGCTTCATTTCCGTCATCT
**XX0488-C.g40 (PCM7d)**	52	E-291F	TCAAGTGGTGAAGAACATGAAGATA
**XX0488-C.g40 (PCM7d)**	52	E-292R	TTGACGTCGGGGAGGTAAATA
**XX0488-C.g40 (PCM7d)**	52	E-293F	ATGAGCGTAATACAGGTGAACCA
**XX0488-C.g40 (PCM7d)**	52	E-294R	TGCGGTACTATAAACGGTGTTACT
**XX0488-C.g40 (PCM7d)**	52	E-295F	ACCTGTGGTTAACTTCTTGTTCGA
**XX0488-C.g40 (PCM7d)**	52	E-296R	ACGCAAACTCCTCGAGATATTCT

**XX0488-C.g40 (PCM7d)**	52	E-297F	TATGGCAAGGAATGTTATGTGCTTT
**XX0488-C.g40 (PCM7d)**	52	E-298R	ACTTTTGGGTATTTGCAGTATTTGGAA
**XX0488-C.g40 (PCM7d)**	52	E-299F	TTATGTTCGATAGCTTTTCTGTTCATGA
**XX0488-C.g40 (PCM7d)**	52	E-300R	TTGTCCAGATCCTCCAAGATAGT
**XX0488-C.g40 (PCM7d)**	52	E-300F	ACTATCTTGGAGGATCTGGACAA

Gene-specific forward primers used in combination with Ex2-reg for Exon 2 PCR

Target	T_A (°C)	Primer name	Sequence 5’-3’
**BC12a**	48	241F	ATGAGGAAAAAGTCAGTGGGAAA
**J1a**	48	310F	ACAACAGATGGAGATAGATATTTATCACT
**J1b**	48	253F	TGAACCAGCAGATAATAGTGATGAA
**J1d**	48	285F	TGGTTAGTTGAATGGGGTAAAGAA
**PCM7a**	48	73F	ATGAAAGAAATATCAGATAAAATAG
**PCM7d**	48	297F	TATGGCAAGGAATGTTATGTGCTTT

DBLβ expression construct primers (contain BamHI (F) and XhoI (R) restriction sites)

Target	T_A (°C)	Primer name	Sequence 5’-3’
**BC12a_DBLβ**	52	E-301F	ggtggaggatccCCATGTGCTAAACCCAGTGGT
**BC12a_DBLβ**	52	E-302R	tccaccctcgagttaACAATTACATGGTGTATCGTGATCAT
**J1a_DBLβ**	52	E-303F	ggtggaggatccGCTTGTAGTGGAGACCCCA
**J1a_DBLβ**	52	E-304R	tccaccctcgagttaACACTTACATACATCACCATACCCA

Supplementary data files

S1 Data. Data for flow adhesion assays (S6 Fig).

S2 Data. Data for SPR experiments for Figs 6D, 6E and 6F.

S3 Data. Data for SPR experiments for Figs 6G and 6H.

S4 Data. Kinetic values for SPR experiments

S5 Data. Data for SPR experiments for S3 Fig.

S6 Data. Data for SPR experiments for S4 Fig.

S7 Data. Data for SPR experiments for S5 Fig.
